# Association of Leukocyte Telomere Length With Mortality Among Adult Participants in 3 Longitudinal Studies

**DOI:** 10.1001/jamanetworkopen.2020.0023

**Published:** 2020-02-26

**Authors:** Konstantin G. Arbeev, Simon Verhulst, Troels Steenstrup, Jeremy D. Kark, Olivia Bagley, Charles Kooperberg, Alexander P. Reiner, Shih-Jen Hwang, Daniel Levy, Annette L. Fitzpatrick, Kaare Christensen, Anatoliy I. Yashin, Abraham Aviv

**Affiliations:** 1 Biodemography of Aging Research Unit, Social Science Research Institute, Duke University, Durham, North Carolina; 2 Groningen Institute for Evolutionary Life Sciences, University of Groningen, the Netherlands; 3 Epidemiology, Biostatistics, and Biodemography, Institute of Public Health, University of South Denmark, Odense, Denmark; 4 Epidemiology Unit, Hebrew University–Hadassah School of Public Health and Community Medicine, Jerusalem, Israel; 5 Division of Public Health Sciences, Fred Hutchinson Cancer Research Center, Seattle, Washington; 6 Department of Epidemiology, University of Washington, Seattle; 7 Population Sciences Branch, National Heart, Lung, and Blood Institute, National Institutes of Health, Bethesda, Maryland; 8 The Framingham Heart Study, Framingham, Massachusetts; 9 Department of Clinical Genetics, Odense University Hospital, Odense, Denmark; 10 Danish Aging Research Center, University of Southern Denmark, Odense, Denmark; 11 Center of Human Development and Aging, New Jersey Medical School, Rutgers, The State University of New Jersey, Newark

## Abstract

**Importance:**

Leukocyte telomere length (LTL) is a trait associated with risk of cardiovascular disease and cancer, the 2 major disease categories that largely define longevity in the United States. However, it remains unclear whether LTL is associated with the human life span.

**Objective:**

To examine whether LTL is associated with the life span of contemporary humans.

**Design, Setting, and Participants:**

This cohort study included 3259 adults of European ancestry from the Cardiovascular Health Study (CHS), Framingham Heart Study (FHS), and Women’s Health Initiative (WHI). Leukocyte telomere length was measured in 1992 and 1997 in the CHS, from 1995 to 1998 in the FHS, and from 1993 to 1998 in the WHI. Data analysis was conducted from February 2017 to December 2019.

**Main Outcomes and Measures:**

Death and LTL, measured by Southern blots of the terminal restriction fragments, were the main outcomes. Cause of death was adjudicated by end point committees.

**Results:**

The analyzed sample included 3259 participants (2342 [71.9%] women), with a median (range) age of 69.0 (50.0-98.0) years at blood collection. The median (range) follow-up until death was 10.9 (0.2-23.0) years in CHS, 19.7 (3.4-23.0) years in FHS, and 16.6 (0.5-20.0) years in WHI. During follow-up, there were 1525 deaths (482 [31.6%] of cardiovascular disease; 373 [24.5%] of cancer, and 670 [43.9%] of other or unknown causes). Short LTL, expressed in residual LTL, was associated with increased mortality risk. Overall, the hazard ratio for all-cause mortality for a 1-kilobase decrease in LTL was 1.34 (95% CI, 1.21-1.47). This association was stronger for noncancer causes of death (cardiovascular death: hazard ratio, 1.28; 95% CI, 1.08-1.52; cancer: hazard ratio, 1.13; 95% CI, 0.93-1.36; and other causes: hazard ratio, 1.53; 95% CI, 1.32-1.77).

**Conclusions and Relevance:**

The results of this study indicate that LTL is associated with a natural life span limit in contemporary humans.

## Key Points

**Question:**

Is leukocyte telomere length associated with the natural life span of contemporary humans?

**Findings:**

This cohort study included 3259 participants from 3 longitudinal studies, of whom 1525 died during the follow-up period. Leukocyte telomere length–associated mortality from noncancer causes increased as participants aged, approaching their age at death.

**Meaning:**

These data suggest that leukocyte telomere length is associated with a life span limit among contemporary humans.

## Teaser

This cohort study examines whether leukocyte telomere length is associated with the life span of contemporary humans.

## Introduction

The debate on the natural life span limit in humans has focused on demographic trends^1,2,3,4,5^ rather than on biological factors that set a ceiling for life span. We hypothesized that leukocyte telomere length (LTL) might be a biological driver of life span because LTL is associated with increased mortality among older individuals^6,7,8,9,10^ and converging evidence infers a causal role of LTL in aging-related diseases that often result in death.^11,12,13,14,15^

The view that LTL plays a causal role in aging-related diseases draws on the following findings. First, LTL variation across individuals as well as some underlying determinants of LTL variation, including high heritability and sex, are similar in newborns and adults.^16^ Second, individuals who enter adult life with short or long LTL are likely to have short or long LTL, respectively, throughout their remaining life course.^17,18^ Therefore, having comparatively short or long LTL is principally determined early in life, typically decades before disease onset and mortality. Third, genome-wide association studies have identified LTL-associated single-nucleotide polymorphisms mapped to several regions that harbor telomere maintenance genes.^11,19,20^ These single-nucleotide polymorphisms have been used to develop genetic risk scores that show an inverse association of LTL with cardiovascular disease (CVD)^11,12,13,21^ and direct associations with some cancers.^12,14,15,21^ Such genetic findings largely exclude reverse causality, ie, that CVD might shorten LTL or some cancers might lengthen LTL. Jointly, these findings suggest that LTL is likely causal for CVD and some cancers, perhaps increasing the mortality risk that arises from these diseases. In addition, based on empirical and theoretical considerations, our previous work showed that a subset of the general population may reach a critically short LTL, a so-called telomeric brink, at an age younger than life expectancy, which denotes a high risk of death in the near term.^22^ The questions are whether LTL is associated with the life span of some individuals and which diseases (ie, CVD, cancer, and other causes [OCs]) might influence such an association. To this end, we leveraged LTL and mortality data from 3 longitudinal studies in the United States.

## Methods

### Sample

Participants of European ancestry with LTL measurements from the Cardiovascular Health Study (CHS),^23^ the Framingham Heart Study (FHS),^24^ and the Women’s Health Initiative (WHI)^25^ were included (eTable 1 in the Supplement). Self-reported race/ethnicity in CHS and WHI was used to select the subsample of participants of European ancestry in these studies. Participants in FHS are almost exclusively self-reported to be of European ancestry. In CHS, LTL was measured in years 5 and 10 (ie, 1992 and 1997); in FHS, at exam 6 (ie, 1995-1998); and in WHI, at exam 1 (ie, 1993-1998). No variables used in the analyses (ie, sex, age at LTL measurement, age at death or censoring, indicators of death events, or LTL) had missing values. Cause of death was adjudicated by end point committees of the respective studies. The CHS used historical data on morbidities, hospitalizations, and medications along with medical records (ie, most recent hospitalization) and proxy interviewers (ie, to ascertain circumstances). These data were sent to the Morbidity and Mortality Committees, which were made up of study physicians from each site, to adjudicate cause of death. The FHS collected detailed information on underlying causes of death (ie, from cardiovascular causes, cancer, other noncardiovascular or noncancer causes, or unknown), performing a comprehensive review of all available medical records by a physician-review, panel-based adjudication process established decades ago. In WHI, mortality events were identified using annual mailings and follow-up (ie, proxy questionnaires, returned mailings) and National Death Index searches. Cause of death was ascertained from death certificates and National Death Index searches by a committee of physician adjudicators.

This study was approved by institutional review board of Rutgers University, the State University of New Jersey. The study performed secondary analyses. No new data were collected from study participants in the course of working on this article. In the original studies, DNA was collected from participants who provided informed consent for genetic research. Study procedures, including obtaining informed consent from study participants, are described in respective publications.^23,24,25^ This report follows the Strengthening the Reporting of Observational Studies in Epidemiology (STROBE) reporting guideline for cohort studies. Data for this study were analyzed from February 2017 to December 2019.

### LTL Measurements

Measurements were performed at a baseline examination by Southern blot of the terminal restriction fragments.^26^ The interassay coefficients of variation were 2.4%, 1.5%, and 2.0% for the FHS, CHS, and WHI, respectively. In the CHS, 963 individuals had LTL measurements from blood samples obtained in year 5. Among those, 612 (63.6%) had a second measurement in year 10. In the analyses reported in this paper, we used the second LTL measurement for the latter group and the first (and only) LTL measurement for the remaining 351 individuals in the analyzed CHS sample. We also performed sensitivity analyses including only the 612 individuals with 2 LTL measurements. These showed qualitatively similar results; therefore, they are not reported here.

### Statistical Analysis

We used *t* tests for comparisons between the LTL of women and men as well as between the LTL of those who died and those who were alive at the end of follow-up. For the former, we used the age- and study-adjusted LTL computed as follows: we regressed LTL on age and study (ie, as a categorical variable with 3 levels, 1 for each study) and added residuals from this regression to the mean LTL across all individuals in the sample. For the latter, we added the residual LTL (rLTL) to the mean LTL across all individuals in the sample. The rLTL was computed as the residuals from linear regressions of LTL on age, fitted separately among women and men in each of the 3 studies. We also fitted the regressions with quadratic terms for age, but these were nonsignificant in all cases; therefore, we proceeded with the linear model. The same values (with added mean LTL in the entire sample) were used in computations of LTL for individuals who died from cancer and noncancerous causes and those alive at the end of follow-up. We also computed the Pearson correlation coefficient between age at blood draw and sex- and study-adjusted LTL (calculated as the residuals from the regression of LTL on sex and the study variable, added to the mean LTL across all individuals in the sample).

We fitted Cox proportional hazards models using follow-up data on mortality in the combined sample. Time since blood draw was used as the time variable. The most parsimonious model included sex and rLTL as covariates. We used 2 flexible specifications to include age in the model: 1 with a natural spline basis for age and another with age included as a linear term, stratified by baseline age, thus allowing for different baseline hazards in each age strata. Both methods showed similar results for the association of rLTL with mortality. In this article, we report results for the model with splines. The results for the second approach appear in eTable 3, eTable 5, eTable 10, eFigure 1, eFigure 2, eFigures 5 to 7, and eFigure 9 in the Supplement. For technical details and a description of sensitivity analyses, see the eAppendix in the Supplement.

We analyzed data on cause-specific mortality in the competing risks context using the cause-specific hazards functions approach.^27^ We used the same model specifications as in the all-cause mortality analyses and estimated respective regression parameters for different cause-specific hazards functions (ie, CVD, OC, and cancer). We report results for the model with splines in the Results section; the results for the second approach appear in eTable 3, eTable 5, eTables 11 to 13, eFigure 1, eFigure 2, eFigure 7, and eFigure 9 in the Supplement. For technical details and description of sensitivity analyses, see the eAppendix in the Supplement.

Statistical analyses with the Cox proportional hazards models and the competing risks models were performed in R version 3.6.1 using the *survival *package (R Project for Statistical Computing). Figures were prepared in MATLAB R2019a (MathWorks) and in R version 3.6.1. Statistical significance was set at *P* < .05, and all tests were 2-tailed.

## Results

The initial sample consisted of 3434 individuals (2439 [68.4%] women and 995 [31.6%] men) of European ancestry, with median (range) age at blood collection of 68.0 (33.0-98.0) years, median (range) age at death of 83.0 (51.0-105.0) years, and a mean (SD) follow-up period of 15.2 (5.4) years (eTable 1 in the Supplement). The median (range) follow-up periods for death events were 10.9 (0.2-23.0) years in CHS, 19.7 (3.4-23.0) years in FHS, and 16.6 (0.5-20.0) years in WHI. We excluded 175 individuals younger than 50 years at the time of blood draw because there were only 13 deaths during follow-up among this subsample (1 [7.7%] of CVD; 3 [23.1%] of OC; 9 [69.2%] of cancer). The analyzed sample included 3259 participants (2342 women [71.9%] and 917 [28.1%] men), with a median (range) age of 69.0 (50.0-98.0) years at the time of blood collection. Participants were followed up until death or were censored at the termination of follow-up for a mean (range) period of 15.0 (0.2-23.0) years. Among eligible participants, 1525 (46.8%) died during the follow-up (482 [31.6%] of CVD; 373 [24.5%] of cancer; and 670 [43.9%] of OCs) (Table).

**Table.** Descriptive Statistics for the Sample Used in Analyses

Characteristic	No. (%)			
	CHS (n = 963)	FHS (n = 1069)	WHI (n = 1227)	Total (N = 3259)
Age, median (range), y	77.0 (66.0-98.0)	60.0 (50.0-86.0)	67.0 (50.0-79.0)	69.0 (50.0-98.0)
Age at death, median (range), y	89.0 (74.0-105.0)	78.6 (54.0-102.0)	82.2 (52.0-98.0)	83.5 (52.0-105.0)
Follow-up period, mean (SD), y	11.2 (5.9)	18.1 (4.2)	15.3 (3.8)	15.0 (5.4)
Women	573 (59.5)	542 (50.7)	1227 (100)	2342 (71.9)
Did not die during follow-up	116 (12.0)	725 (67.8)	893 (72.8)	1734 (53.2)
Died during follow-up	847 (88.0)	344 (32.2)	334 (27.2)	1525 (46.8)
CVD	292 (30.3)	81 (7.6)	109 (8.9)	482 (14.8)
OC	412 (42.8)	146 (13.7)	112 (9.1)	670 (20.6)
Cancer	143 (14.8)	117 (10.9)	113 (9.2)	373 (11.4)
Aged 50-60 y	0	3 (0.3)	3 (0.2)	6 (0.2)
Aged 61-70 y	0	39 (3.6)	43 (3.5)	82 (2.5)
Aged 71-80 y	71 (7.4)	113 (10.6)	114 (9.3)	298 (9.1)
Aged >80 y	776 (80.6)	189 (17.7)	174 (14.2)	1139 (34.9)

Abbreviations: CHS, the Cardiovascular Health Study; CVD, cardiovascular disease; FHS, the Framingham Heart Study; OC, other causes (ie, not cancer or CVD); WHI, Women’s Health Initiative.

**Figure 1.**
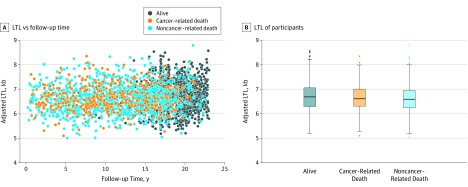
Leukocyte Telomere Length (LTL) and Mortality B, The bottom and top of each box indicate the first and third quartiles, respectively. The points outside the whiskers represent outlying observations beyond 1.5 times the interquartile range. With Bonferroni adjustment, *P* = .75 for alive vs cancer-related death, *P* < .001 for alive vs noncancer-related death, and *P* = 0.49 for cancer-related death vs noncancer-related death. Kb indicates kilobase.

Sex- and study-adjusted LTL was inversely correlated with age (*r* = −0.20; *P* < .001; slope [SE], −0.012 [0.001]). Men had shorter mean (SD) age- and study-adjusted LTL than women (6.58 [0.51] kilobase [kb] vs 6.69 [0.56] kb; *P* < .001). Age-, sex-, and study-adjusted LTL vs follow-up time after blood draw is displayed in Figure 1A. Participants who survived to the end of follow-up showed a significantly longer median LTL compared with those who died from noncancer causes but compared with those who died from cancer (alive: 6.69 kb; 95% CI, 6.66-6.72 kb; cancer-related death: 6.61 kb; 95% CI, 6.55-6.67 kb; noncancer-related death: 6.59 kb; 95% CI, 6.56-6.62 kb) (Figure 1B).

**Figure 2.**
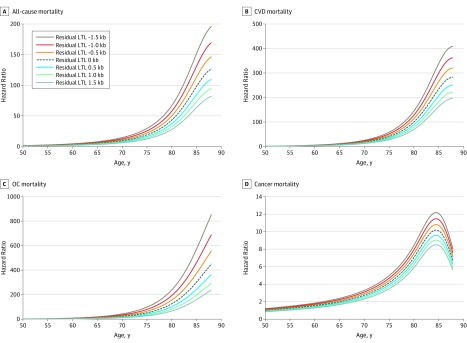
Hazard Ratios for All-Cause and Cause-Specific Mortality for Different Residual Leukocyte Telomere Lengths (LTLs) Hazard ratios for individuals of different ages and with different residual LTLs were scaled to an individual aged 50 years with the mean LTL value for that age (ie, residual LTL of 0). Lines are truncated at the age corresponding to the 99th percentile of the distribution of ages at blood draw in the sample. When using flexible spline modeling, the SEs of the curves are largest in the tails of the distribution, which may explain why the curves for cancer mortality (D) turn downwards. The graphs in this figure are the point estimates and ignore uncertainty. However, a continued increase of the curves for high ages cannot be ruled out based on the model fit. Kb indicates kilobase.

**Figure 3.**
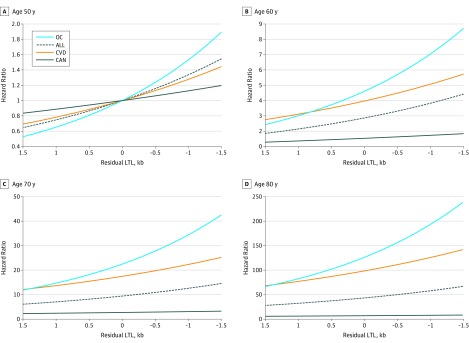
Hazard Ratios for All-Cause and Cause-Specific Mortality at Different Ages Hazard ratios for individuals of different ages and with different values of residual leukocyte telomere length (LTL) were scaled to an individual aged 50 years with the expected mean LTL for that age (ie, residual LTL of 0). ALL indicates all-cause mortality; CAN, cancer mortality; CVD, cardiovascular disease mortality; kb, kilobase; and OC, other causes of death.

Figure 2 displays the hazards of death from all causes, CVD, OCs, and cancer among individuals of different ages and with different values of rLTL, scaled to an individual aged 50 years with the expected (ie, mean) value of LTL for that age (ie, rLTL of 0). Figure 3 shows the respective hazards of death from different causes at specific ages for different values of rLTLs. At age 60 years, HRs were higher for an individual with −1.0 kb rLTL compared with an individual with 0 kb rLTL (all-cause mortality: 3.8 [95% CI, 3.2-4.6] vs 2.9 [95% CI, 2.4-3.4]; CVD mortality: 5.1 [95% CI, 3.5-7.3] vs 4.0 [95% CI, 2.9-5.5]; OC mortality: 7.1 [95% CI, 5.1-9.7] vs 4.6 [95% CI, 3.5-6.1]). At age 80 years, HRs were higher for an individual with −1.0 kb rLTL compared with an individual with 0 kb rLTL (all-cause mortality: 57.7 [95% CI, 43.5-76.7] vs 43.2 [95% CI, 33.2-56.2]; CVD mortality: 125.5 [95% CI, 69.6-226.3] vs 98.3 [95% CI, 56.2-171.9]; OC mortality: 192.6 [95% CI, 115.5-321.3] vs 125.9 [95% CI, 77.5-204.4]) (eTables 6-8 in the Supplement).

However, we observed minimal association of LTL with cancer mortality (Figure 2D; eTable 9 in the Supplement). At age 60 years, an individual with 0 kb rLTL had an HR of 1.5 (95% CI, 1.2-2.0), while an individual with −1.0 kb rLTL had an HR of 1.7 (95% CI, 1.3-2.4). At age 80 years, an individual with 0 kb rLTL had an HR of 7.0 (95% CI, 4.6-10.4), while an individual with −1.0 kb rLTL had an HR of 7.8 (95% CI, 5.0-12.4).

For this reason, we repeated analyses excluding all cancer-related mortality. The sample for this analysis contained 2886 individuals, ie, all individuals in the sample used in analyses shown in Figure 2 and Figure 3, excluding 373 individuals who died of cancer during the follow-up period (eFigures 3-6 in the Supplement). At age 60 years, an individual with 0 kb rLTL had an HR of 5.0 (95% CI, 4.1-6.2), while an individual with −1.0 kb rLTL had an HR of 7.0 (95% CI, 5.5-9.0). At age 80 years, an individual with 0 kb rLTL had an HR of 147.8 (95% CI, 102.6-213.1), while an individual with −1.0 kb rLTL had an HR of 207.9 (95% CI, 141.3-306.0).

**Figure 4.**
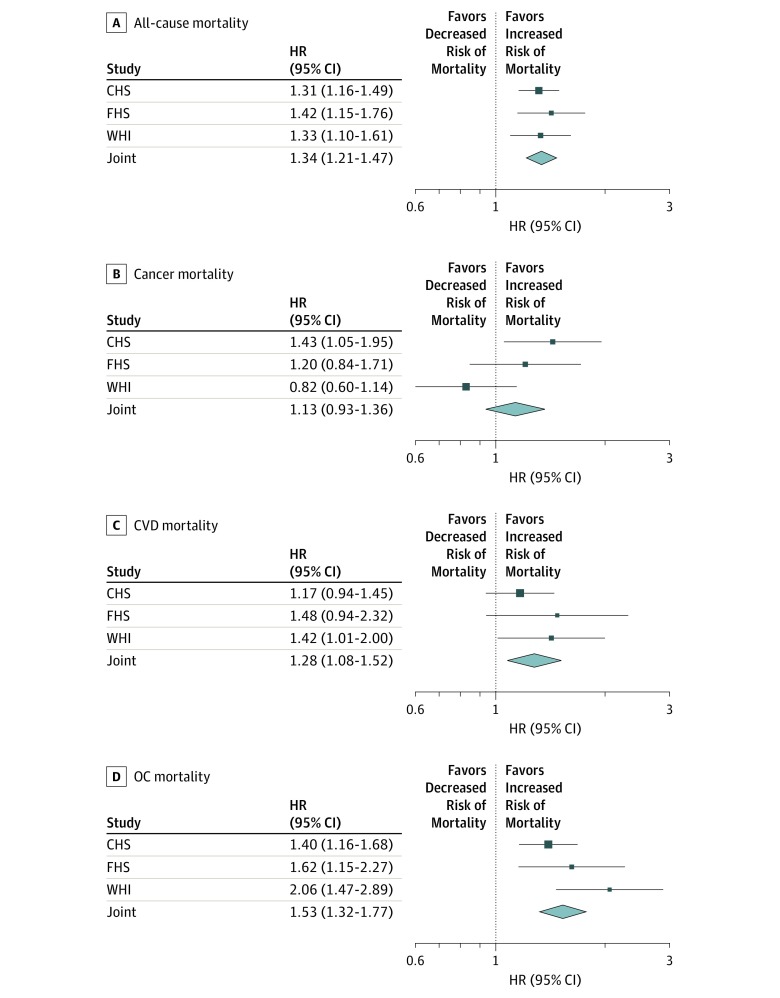
Hazard Ratios (HRs) for Residual Leukocyte Telomere Length in Different Studies and Joint Analysis The figure displays HRs for a 1-kilobase decrease in LTL, estimated in respective models, applied to separate studies and joint analyses. Squares indicate the effect sizes for each study, with lines representing the 95% CIs. Estimates for joint analyses and their 95% CIs are represented by diamonds. CHS indicates the Cardiovascular Health Study; CVD, cardiovascular disease; FHS, the Framingham Heart Study, OC, other cause; and WHI, the Women’s Health Initiative.

We also performed analyses in separate samples (ie, CHS, FHS, WHI) (eAppendix, eTable 4, and eTable 5 in the Supplement). Figure 4 shows the HRs for a 1-kb decrease in rLTL for each study individually and jointly (all-cause mortality in CHS: HR, 1.31; 95% CI, 1.16-1.49; FHS: 1.42; 95% CI, 1.15-1.76; WHI: 1.33; 95% CI, 1.10-1.61; joint: HR, 1.34; 95% CI, 1.21-1.47). All HRs displayed the same direction in all 3 studies as in the joint analyses, except for the HR for cancer in WHI, which showed the opposite direction as the other studies (cancer mortality in CHS: HR, 1.43; 95% CI, 1.05-1.95; FHS: 1.20; 95% CI, 0.84-1.71; WHI: HR, 0.82; 95% CI, 0.60-1.14; joint: HR, 1.13; 95% CI, 0.93-1.36). The joint HR for CVD mortality was 1.28 (95% CI, 1.08-1.52) and, for OC mortality, 1.53 (95% CI, 1.32-1.77). Similar observations also held for an alternative modeling with age strata (eFigure 7 in the Supplement).

Given that different studies had different durations of follow-up (Table), we repeated all calculations, truncating follow-up at 15 years (ie, approximately the mean follow-up in the sample). All results were similar to those presented in the text (eFigure 8 and eFigure 9 in the Supplement).

We found evidence of selection among individuals older than 80 years (variances in the groups aged <80 years vs ≥80 years: 0.40 and 0.29; Levene test for equality of variances for the groups: *P* < .001) (eFigure 10 in the Supplement). This selection is likely owing in part to the earlier deaths among individuals who died from cancer, given that the median age at death from cancer in the analyzed sample was younger than the median age of death from CVD or OCs (81.0 years vs 86.6 years; Kruskal-Wallis test for equality of medians in these groups: *P* < .001), in line with findings in the general US population.^28,29^ Consistent with this premise, rLTLs for individuals who died of cancer at ages younger than 80 years were longer than the rLTLs for those who died of noncancer causes at the same age (6.65 kb vs 6.51 kb; *P* = .02).

Models in which age was included as a linear term, stratified by baseline age, allowed for different baseline hazards in each age strata. These had similar results as the main analysis (eAppendix, eTables 2-5, eFigure 1, and eFigure 2 in the Supplement).

## Discussion

The debate among demographers on the natural life span limit in humans detracts from a more persistent question about biological factors that may determine such a limit. The potential roles of these factors must be considered in the context of specific causes of death. This study showed that short LTL was associated with increased mortality risk as individuals approached the upper boundary of their longevity, a phenomenon principally associated with mortality from noncancer causes.

In absolute terms, the HRs associated with short LTL rapidly escalated as an individual’s age approached 90 years. Simply stated, an LTL-associated increased mortality risk from 100 to 150 in an individual approaching the upper boundary of the human life span is not akin to an LTL-associated increased mortality risk from 1.0 to 1.5 at a much younger age. Ultimately, the estimates from the models (ie, parameters or corresponding HRs) translate to the estimates of survival probabilities (or probabilities of death) for individuals with different rLTLs at different ages. At younger ages, the mortality risk is low (ie, the survival probabilities are close to 1) such that the resulting change in survival probabilities would not be substantial. However, at older ages, the mortality risk is much higher by itself (ie, the survival probabilities are small and approach 0 as age increases). This means that survival chances associated with shorter LTLs among older individuals are dramatically lower for the same relative increase in the risk of death as at younger ages. While an individual with an rLTL of 0 kb at age 85 years might have a small but still noticeable survival probability, for an individual aged 85 years with shorter rLTL (eg, −1 kb), such probability might become considerably smaller. This concept might hold not only for LTL but also for other aging-related phenotypes, whose presumed association with survival probability increases in absolute terms as individuals approach the boundary of the human life span.

Given common misclassifications of cause of death based on death certificates,^30,31^ accurate determination of the cause of death was critical for our conclusion that LTL was more strongly associated with death from noncancer causes than death from cancer. That said, death among the older individuals, even when carefully adjudicated, is often not a consequence of a single disease. For instance, stroke or myocardial infarction may occur in different clinical settings among individuals who have multiple health problems (eg, frailty, loss of ambulation due to a fall, diabetes, dementia, infection, etc) that collectively contribute to the individual’s death. Regardless of these specific circumstances, it is clear that having comparatively short LTL was associated with increased mortality risk from noncancer causes (ie, CVD and OCs).

Regarding the minimal association of LTL with cancer mortality, we note that, whereas comparatively long LTL^32,33,34^ and alleles associated with a long LTL^12,14,15^ have been reported to be associated with increased risk of several cancers, short LTL has been reported to be associated with diminished survival among patients with some but not all cancers.^35,36,37,38^ Hence, the association of LTL with cancer mortality is complex and contextual; it may reflect opposing telomere-related elements that modify cancer risk, outcome of cancer treatment, and survival.

### Limitations

This study has limitations. Our findings are based on individuals of European ancestry who reside in the United States. These results therefore need replication in other groups and geographic locations, given that there is some evidence that the association of LTL with mortality might be influenced by ethnicity.^25^ In addition, our analyses did not adjust for key risk factors that are associated with mortality risk (eg, hypertension, dyslipidemia, diabetes, smoking, obesity) or for comorbidities that may have been present at baseline and also contributed to mortality (eg, CVD and cancer).

## Conclusions

In this study, comparatively short LTL was associated with an increased risk of dying from noncancer causes in absolute terms among individuals as they approached the upper boundary of human longevity. Further research is needed to assess whether a causal relationship exists and to determine the contribution of LTL to the natural life span limit in contemporary humans.

## Notes

Supplement. **eAppendix. **Technical Details **eTable 1.** Descriptive Statistics for the Original Sample **eTable 2.** Results of Analyses of All-Cause and Cause-Specific Mortality in the Combined Sample of 3 Studies (CHS, FHS, WHI) Using the Model With Splines for Age **eTable 3.** Results of Analyses of All-Cause and Cause-Specific Mortality in the Combined Sample of 3 Studies (CHS, FHS, WHI) Using the Model With Stratified Baseline Hazards for Age Groups **eTable 4.** Results of Analyses of All-Cause and Cause-Specific Mortality in Separate Samples (CHS, FHS, WHI) Using the Model With Splines for Age **eTable 5.** Results of Analyses of All-Cause and Cause-Specific Mortality in Separate Samples (CHS, FHS, WHI) Using the Model With Stratified Baseline Hazards for Age Groups **eTable 6.** Hazard Ratios for All-Cause Mortality for Different Ages and Residual Leukocyte Telomere Length (rLTL) Computed From the Cox Model With Splines for Age **eTable 7.** Hazard Ratios for Cardiovascular Disease Mortality for Different Ages and Residual Leukocyte Telomere Length (rLTL) Computed From the Cox Model With Splines for Age **eTable 8.** Hazard Ratios for Other Causes Mortality for Different Ages and Residual Leukocyte Telomere Length (rLTL) Computed From the Cox Model With Splines for Age **eTable 9.** Hazard Ratios for Cancer Mortality for Different Ages and Residual Leukocyte Telomere Length (rLTL) Computed From the Cox Model With Splines for Age **eTable 10.** Hazard Ratios for All-Cause Mortality for Different Ages and Residual Leukocyte Telomere Length (rLTL) Computed From the Cox Model With Stratified Baseline Hazards for Age Groups **eTable 11.** Hazard Ratios for Cardiovascular Disease Mortality for Different Ages and Residual Leukocyte Telomere Length (rLTL) Computed From the Cox Model With Stratified Baseline Hazards for Age Groups **eTable 12.** Hazard Ratios for Other Causes Mortality for Different Ages and Residual Leukocyte Telomere Length (rLTL) Computed From the Cox Model With Stratified Baseline Hazards for Age Groups **eTable 13.** Hazard Ratios for Cancer Mortality for Different Ages and Residual Leukocyte Telomere Length (rLTL) Computed From the Cox Model With Stratified Baseline Hazards for Age Groups **eFigure 1.** Hazard Ratios for All-Cause and Cause-Specific Mortality for Different Residual Leukocyte Telomere Lengths **eFigure 2.** Hazard Ratios for All-Cause and Cause-Specific Mortality at Different Ages **eFigure 3.** Hazard Ratios for Noncancer Mortality for Different Ages and Values of Residual Leukocyte Telomere Length **eFigure 4.** Hazard Ratios at Different Ages for Noncancer Mortality in the Model with Splines for Age **eFigure 5.** Hazard Ratios for Noncancer Mortality for Different Ages and Values of Residual Leukocyte Telomere Length **eFigure 6.** Hazard Ratios at Different Ages for Noncancer Mortality in the Model With Stratified Baseline Hazards **eFigure 7.** Hazard Ratios for Residual Leukocyte Telomere Length in Different Studies and Joint Analysis in the Model With Stratified Baseline Hazards **eFigure 8.** Hazard Ratios for Residual Leukocyte Telomere Length in Different Studies and Joint Analysis With Truncated Follow-up in the Model With Splines **eFigure 9.** Hazard Ratios for Residual Leukocyte Telomere Length in Different Studies and Joint Analysis With Truncated Follow-up in the Model With Stratified Baseline Hazards **eFigure 10.** Density Plots of Leukocyte Telomere Length for Different Age Groups
