# Supplementary material for: Association of Leukocyte Telomere Length With Mortality Among Adult Participants in 3 Longitudinal Studies
Source: JAMA Netw Open. 2020 Feb 26;3(2):e200023. doi: 10.1001/jamanetworkopen.2020.0023 (PMC7137690; doi:10.1001/jamanetworkopen.2020.0023)
Supplement: Supplement. — eAppendix. Technical Details eTable 1. Descriptive Statistics for the Original Sample eTable 2. Results of Analyses of All-Cause and Cause-Specific Mortality in the Combined Sample of 3 Studies (CHS, FHS, WHI) Using the Model With Splines for Age eTable 3. Results of Analyses of All-Cause and Cause-Specific Mortality in the Combined Sample of 3 Studies (CHS, FHS, WHI) Using the Model With Stratified Baseline Hazards for Age Groups eTable 4. Results of Analyses of All-Cause and Cause-Specific Mortality in Separate Samples (CHS, FHS, WHI) Using the Model With Splines for Age eTable 5. Results of Analyses of All-Cause and Cause-Specific Mortality in Separate Samples (CHS, FHS, WHI) Using the Model With Stratified Baseline Hazards for Age Groups eTable 6. Hazard Ratios for All-Cause Mortality for Different Ages and Residual Leukocyte Telomere Length (rLTL) Computed From the Cox Model With Splines for Age eTable 7. Hazard Ratios for Cardiovascular Disease Mortality for Different Ages and Residual Leukocyte Telomere Length (rLTL) Computed From the Cox Model With Splines for Age eTable 8. Hazard Ratios for Other Causes Mortality for Different Ages and Residual Leukocyte Telomere Length (rLTL) Computed From the Cox Model With Splines for Age eTable 9. Hazard Ratios for Cancer Mortality for Different Ages and Residual Leukocyte Telomere Length (rLTL) Computed From the Cox Model With Splines for Age eTable 10. Hazard Ratios for All-Cause Mortality for Different Ages and Residual Leukocyte Telomere Length (rLTL) Computed From the Cox Model With Stratified Baseline Hazards for Age Groups eTable 11. Hazard Ratios for Cardiovascular Disease Mortality for Different Ages and Residual Leukocyte Telomere Length (rLTL) Computed From the Cox Model With Stratified Baseline Hazards for Age Groups eTable 12. Hazard Ratios for Other Causes Mortality for Different Ages and Residual Leukocyte Telomere Length (rLTL) Computed From the Cox Model With Stratified Baseline Hazards for Age Groups e [file jamanetwopen-3-e200023-s001.pdf]

Supplementary Online Content

Arbeev KG, Verhulst S, Steenstrup T, et al. Association of leukocyte telomere length with mortality among adult participants in 3 longitudinal studies. *JAMA Netw Open*. 2020;3(2):e200023. doi:10.1001/jamanetworkopen.2020.0023

eAppendix. Technical Details

eTable 1. Descriptive Statistics for the Original Sample

eTable 2. Results of Analyses of All-Cause and Cause-Specific Mortality in the Combined Sample of 3 Studies (CHS, FHS, WHI) Using the Model With Splines for Age

eTable 3. Results of Analyses of All-Cause and Cause-Specific Mortality in the Combined Sample of 3 Studies (CHS, FHS, WHI) Using the Model With Stratified Baseline Hazards for Age Groups

eTable 4. Results of Analyses of All-Cause and Cause-Specific Mortality in Separate Samples (CHS, FHS, WHI) Using the Model With Splines for Age

eTable 5. Results of Analyses of All-Cause and Cause-Specific Mortality in Separate Samples (CHS, FHS, WHI) Using the Model With Stratified Baseline Hazards for Age Groups

eTable 6. Hazard Ratios for All-Cause Mortality for Different Ages and Residual Leukocyte Telomere Length (rLTL) Computed From the Cox Model With Splines for Age

eTable 7. Hazard Ratios for Cardiovascular Disease Mortality for Different Ages and Residual Leukocyte Telomere Length (rLTL) Computed From the Cox Model With Splines for Age

eTable 8. Hazard Ratios for Other Causes Mortality for Different Ages and Residual Leukocyte Telomere Length (rLTL) Computed From the Cox Model With Splines for Age

eTable 9. Hazard Ratios for Cancer Mortality for Different Ages and Residual Leukocyte Telomere Length (rLTL) Computed From the Cox Model With Splines for Age

eTable 10. Hazard Ratios for All-Cause Mortality for Different Ages and Residual Leukocyte Telomere Length (rLTL) Computed From the Cox Model With Stratified Baseline Hazards for Age Groups

eTable 11. Hazard Ratios for Cardiovascular Disease Mortality for Different Ages and Residual Leukocyte Telomere Length (rLTL) Computed From the Cox Model With Stratified Baseline Hazards for Age Groups

eTable 12. Hazard Ratios for Other Causes Mortality for Different Ages and Residual Leukocyte Telomere Length (rLTL) Computed From the Cox Model With Stratified Baseline Hazards for Age Groups

eTable 13. Hazard Ratios for Cancer Mortality for Different Ages and Residual Leukocyte Telomere Length (rLTL) Computed From the Cox Model With Stratified Baseline Hazards for Age Groups

eFigure 1. Hazard Ratios for All-Cause and Cause-Specific Mortality for Different Residual Leukocyte Telomere Lengths

© 2020 Arbeev KG et al. *JAMA Network Open.*

eFigure 2. Hazard Ratios for All-Cause and Cause-Specific Mortality at Different Ages

eFigure 3. Hazard Ratios for Noncancer Mortality for Different Ages and Values of Residual Leukocyte Telomere Length

eFigure 4. Hazard Ratios at Different Ages for Noncancer Mortality in the Model with Splines for Age

eFigure 5. Hazard Ratios for Noncancer Mortality for Different Ages and Values of Residual Leukocyte Telomere Length

eFigure 6. Hazard Ratios at Different Ages for Noncancer Mortality in the Model With Stratified Baseline Hazards

eFigure 7. Hazard Ratios for Residual Leukocyte Telomere Length in Different Studies and Joint Analysis in the Model With Stratified Baseline Hazards

eFigure 8. Hazard Ratios for Residual Leukocyte Telomere Length in Different Studies and Joint Analysis With Truncated Follow-up in the Model With Splines

eFigure 9. Hazard Ratios for Residual Leukocyte Telomere Length in Different Studies and Joint Analysis With Truncated Follow-up in the Model With Stratified Baseline Hazards

eFigure 10. Density Plots of Leukocyte Telomere Length for Different Age Groups

This supplementary material has been provided by the authors to give readers additional information about their work.

© 2020 Arbeev KG et al. *JAMA Network Open.*

eAppendix. Technical Details

This note provides details of the Cox regression models including details about testing of the model assumption and sensitivity analyses.

Cox regression models. We fitted Cox proportional hazards models using follow-up data on mortality in the combined sample. Time since blood draw was used as the time variable. In the CHS sample, it is time since either year 5 (for those with one LTL measurement) or year 10 (for a subset with the second LTL measurement). The model included sex and rLTL as covariates.

We used two flexible specifications to include age (at blood draw) in the model. First, we included age as a linear term and assumed different baseline hazards in age strata:

$$\mu(t,s,rLTL,sex,a_0)=\mu_{0,s}(t)e^{\beta_{rLTL}rLTL+\beta_{sex}sex+\beta_{a_0}(a_0-50)} \quad (1)$$

where t is time since blood draw, $\mu_{0,s}(t)$ is the baseline hazard in age strata s; a_0 is age at blood draw; rLTL is the LTL computed as described in section "Calculation of the residual LTL"; sex is a binary variable coded as 1 – male, 0 – female. The respective competing risks model have similar specifications for cause-specific hazards functions as in eq. (1). We performed all computations with 5-year and 10-year age groups for strata. We combined younger and older age groups to have reasonable numbers of events in the resulting strata. Specifically, we used the following two sets of age groups for strata: <75, 75-79, and 80+; <70, 70-79, and 80+. Both versions showed similar results. Therefore, we report only those with the latter version because it has larger numbers of individuals and events in the middle stratum.

The second approach used a smooth function of age defined by spline basis functions for age in the model:

$$\mu(t,rLTL,sex,a_0)=\mu_0(t)e^{\beta_{rLTL}rLTL+\beta_{sex}sex+\sum_{k=1}^{n_K}\beta_{sb_k}sb_k(a_0)} \quad (2)$$

where sb_k is k^{th} spline basis function and β_{sb_k} is its respective regression coefficient. We generated the respective B-spline basis matrix for natural cubic splines using the *ns*() function from the R-package *splines*. To impose the natural boundary conditions and anchor the B-spline basis, we used the smallest and largest values for age at death in the data (52.74 and 104.07 years) as boundary knots. Initially, we ran the analyses with five basis functions ($n_K=5$) with four interior knots put roughly equidistantly on the quantile scale for uncensored data (that is, corresponding to the 20th, 40th, 60th, and 80th percentiles of the distribution of ages at death in the combined sample: 78, 83, 87, and 91 years, respectively). The analyses revealed a singularity between the 4th and 5th spline basis functions which were identical through the 3rd interior knot. Therefore, we opted for using the smaller number ($n_K=3$) of basis functions with two interior knots at ages corresponding to the 33.33 and 66.67 percentiles of the distribution of ages at death in the combined sample (82 and 88 years, respectively). Nevertheless, the results of analyses with three and five basis functions were nearly identical. We report those with $n_K=3$ in the main text. The respective competing risks model have similar specifications for cause-specific hazards functions as in eq. (2) with basis functions and knots specified as described above.

R codes performing analyses in models (1) and (2) are available in GitHub (https://github.com/okb1/rLTL).

Figures 2-3 in the main text (as well as eFigures 1-6 and eTables 6-13 in the Supplement) display the hazard ratios (HRs) from respective models specified by eqs. (1)-(2) for different ages and rLTLs (-1.5 to +1.5 kb) compared to a 50-year old individual with zero rLTL. In the model with splines, the respective quantities are given by $e^{\beta_{rLTL}rLTL+\sum_{k=1}^{n_K}\beta_{sb_k}(sb_k(a_0)-sb_k(50))}$ for respective ages (a_0) and rLTLs. In the model with age strata, the respective baseline hazards do not cancel out for strata other than the first one containing age 50. Therefore, the respective quantities $e^{\beta_{rLTL}rLTL+\beta_{a_0}(a_0-50)}$ need to be multiplied by respective ratios of baseline hazards for other strata. To estimate such ratios, we fitted the baseline survival functions in respective analyses (using the output from the R-package *survival*) in each strata by the Gompertz functions and used the fitted Gompertz parameters (a_s and b_s) to estimate the baseline hazards $\mu_{0,s}(t)=a_s e^{b_s t}$. In all strata and all analyses, the Gompertz functions provided a good fit to the estimated baseline survival functions (adjusted R^2 were larger than 0.985 in all cases).

We also performed analyses with additional specifications of the Cox models. We fitted the models with the quadratic terms for rLTL as well as those with the interaction terms for rLTL and sex with age (in the stratified model). The estimated parameters (the quadratic term for rLTL and the interaction terms) were not significant. Therefore, we report the more parsimonious specification of the model as described above.

Proportionality of hazards assumption. We tested the proportionality of hazards assumption for covariates in the Cox models using the test based on Schoenfeld residuals implemented in the R-package *survival*. The results are shown in column "P_{PH}" in respective tables. As seen in eTables 2-3 in the Supplement, respective p-values were larger than 0.05 for all all-cause, cardiovascular (CVD) and other causes (OC) mortality thus indicating that the use of the proportional hazards models was justified. For cancer mortality, the proportionality of hazards assumption holds for all variables except sex in the model with splines, thus adding to the complex story about cancer mortality presented in the main text and Supplement.

Analyses in separate studies and sensitivity analyses removing cancer mortality. We also performed analyses in separate samples (CHS, FHS, WHI; Figure 4, eFigure 7, eTables 4-5 in the Supplement). The estimates for the rLTL coefficients for all-cause mortality are negative and significant in all studies (as in the combined analyses). As column "P_{PH}", eTables 4-5 in the Supplement indicates, for separate studies the situation with the proportionality of hazards assumption for all-cause mortality is more complex. While in FHS p-values exceed 0.05 for all covariates, for some covariates in CHS and WHI $p<0.05$, thus indicating these results should be viewed with caution. Column "P_{PH}", eTables 4-5 in the Supplement confirms that the proportionality of hazards assumptions holds for cause-specific mortality models in all these studies, except for age for cancer mortality in CHS in the model with stratified baseline hazards. The HRs for CVD and OC in each dataset follow the same pattern as in the combined analysis (Figure 4; eFigure 7 in the Supplement). The HR for cancer in CHS and FHS follow the pattern in the combined analysis but it is different for WHI, which focuses on women only. Moreover, women with history of self-reported myocardial infarction or coronary revascularization prior to blood collection were excluded from the WHI telomere study. However, the estimate for the rLTL coefficient for cancer in WHI is non-significant (similarly to the combined analysis). As the observed association of LTL with cancer mortality is complex, we performed analyses excluding all cancer-related mortality. The results are shown in eFigures 3-6 in the Supplement. The test of the proportionality of hazards assumption for this analysis indicated that the use of the proportional hazards models was justified in this case ($p>0.05$ for all parameters in both modelling approaches).

© 2020 Arbeev KG et al. *JAMA Network Open.*

eTable 1. Descriptive Statistics for the Original Sample

	CHS	FHS	WHI	Total
Total number of participants	963	1244	1227	3434
Age, median (range)	77 (66-98)	58 (33-86)	67 (50-79)	68 (33-98)
Age at death, median (range)	89.0 (74-105)	77.0 (51-102)	82.2 (52-98)	83.0 (51-105)
Follow-up period, mean (SD)	11.2 (5.9)	18.3 (4.0)	15.3 (3.8)	15.2 (5.4)
Women, No. (%)	573 (59.5)	639 (51.4)	1227 (100)	2439 (71)
Participants who died during the follow-up, No. (%)	847 (88)	357 (28.7)	334 (27.2)	1538 (44.8)
Participants who have not died during the follow-up, No. (%)	116 (12)	887 (71.3)	893 (72.8)	1896 (55.2)
Participants who died from CVD, No. (%)	292 (30.3)	82 (6.6)	109 (8.9)	483 (14.1)
Participants who died from OC, No. (%)	412 (42.8)	149 (12)	112 (9.1)	673 (19.6)
Participants who died from cancer, No. (%)	143 (14.8)	126 (10.1)	113 (9.2)	382 (11.1)
Participants who died at ages 50-60 years, No. (%)	0 (0)	13 (1)	3 (0.2)	16 (0.5)
Participants who died at ages 61-70 years, No. (%)	0 (0)	42 (3.4)	43 (3.5)	85 (2.5)
Participants who died at ages 71-80 years, No. (%)	71 (7.4)	113 (9.1)	114 (9.3)	298 (8.7)
Participants who died at ages >80 years, No. (%)	776 (80.6)	189 (15.2)	174 (14.2)	1139 (33.2)
Participants aged <50 years at the time of blood draw	0	175	0	175
Participants aged <50 years at the time of blood draw who died during the follow-up	0	13	0	13
Participants aged <50 years at the time of blood draw who died from CVD	0	1	0	1
Participants aged <50 years at the time of blood draw who died from OC	0	3	0	3
Participants aged <50 years at the time of blood draw who died from cancer	0	9	0	9

Notes: CHS: the Cardiovascular Health Study; FHS: the Framingham Heart Study; WHI: the Women's Health Initiative; CVD: cardiovascular diseases; OC: other causes (not cancer or CVD). Individuals with history of self-reported myocardial infarction or coronary revascularization prior to blood collection had been excluded in the LTL study of the WHI.

eTable 2. Results of Analyses of All-Cause and Cause-Specific Mortality in the Combined Sample of 3 Studies (CHS, FHS, WHI) Using the Model With Splines for Age

Cause	Covariate	Regression Coefficient	St. Err.	P-value	HR (95% C.I.)	P_{PH}
all-cause	rLTL	-0.2901	0.0490	3.3E-09	1.34 (1.21, 1.47)	0.13
all-cause	sex	0.3085	0.0545	1.5E-08	1.36 (1.22, 1.51)	0.08
CVD	rLTL	-0.2445	0.0875	0.0052	1.28 (1.08, 1.52)	0.41
CVD	sex	0.3453	0.0963	0.0003	1.41 (1.17, 1.71)	0.50
OC	rLTL	-0.4257	0.0752	1.5E-08	1.53 (1.32, 1.77)	0.15
OC	sex	0.2727	0.0828	0.001	1.31 (1.12, 1.55)	0.61
cancer	rLTL	-0.1197	0.0965	0.22	1.13 (0.93, 1.36)	0.36
cancer	sex	0.3409	0.1098	0.002	1.41 (1.13, 1.74)	0.04

Notes: rLTL is residual leukocyte telomere length computed as described in Methods; sex is a binary variable coded as 1 – male, 0 – female; HR denotes hazard ratios (per 1 kb *decrease* for rLTL) with 95% confidence intervals in parentheses; column "P-value" shows p-values for testing the null hypothesis that respective regression coefficient is zero; column "P_{PH}" shows p-values for testing the proportionality of hazards assumption (using the test based on Schoenfeld residuals implemented in the R package "survival"); CVD: cardiovascular diseases; OC: other causes (not cancer or CVD).

© 2020 Arbeev KG et al. *JAMA Network Open.*

eTable 3. Results of Analyses of All-Cause and Cause-Specific Mortality in the Combined Sample of 3 Studies (CHS, FHS, WHI) Using the Model With Stratified Baseline Hazards for Age Groups

Cause	Covariate	Regression Coefficient	St. Err.	P-value	HR (95% C.I.)	P_{PH}
all-cause	a_0^*	0.1139	0.0071	5.2E-58	1.12 (1.11, 1.14)	0.92
all-cause	rLTL	-0.2856	0.0490	5.4E-09	1.33 (1.21, 1.46)	0.20
all-cause	sex	0.3132	0.0546	9.6E-09	1.37 (1.23, 1.52)	0.13
CVD	a_0^*	0.1334	0.0133	7.6E-24	1.14 (1.11, 1.17)	0.81
CVD	rLTL	-0.2348	0.0871	0.007	1.26 (1.07, 1.50)	0.60
CVD	sex	0.3323	0.0966	0.0006	1.39 (1.15, 1.68)	0.54
OC	a_0^*	0.1556	0.0115	5.1E-42	1.17 (1.14, 1.19)	0.60
OC	rLTL	-0.4217	0.0754	2.2E-08	1.52 (1.32, 1.77)	0.15
OC	sex	0.2762	0.0830	0.0009	1.32 (1.12, 1.55)	0.66
cancer	a_0^*	0.0446	0.0121	0.0002	1.05 (1.02, 1.07)	0.29
cancer	rLTL	-0.1125	0.0963	0.24	1.12 (0.93, 1.35)	0.46
cancer	sex	0.3266	0.1099	0.003	1.39 (1.12, 1.72)	0.08

Notes: $a_0^* = a_0 - 50$, a_0 is age at blood draw; rLTL is residual leukocyte telomere length computed as described in Methods; sex is a binary variable coded as 1 – male, 0 – female; HR denotes hazard ratios (per 1 year for age and 1 kb *decrease* for rLTL) with 95% confidence intervals in parentheses; column "P-value" shows p-values for testing the null hypothesis that respective regression coefficient is zero; column "P_{PH}" shows p-values for testing the proportionality of hazards assumption (using the test based on Schoenfeld residuals implemented in the R package "survival"); CVD: cardiovascular diseases; OC: other causes (not cancer or CVD).

© 2020 Arbeev KG et al. *JAMA Network Open.*

eTable 4. Results of Analyses of All-Cause and Cause-Specific Mortality in Separate Samples (CHS, FHS, WHI) Using the Model With Splines for Age

Study	Cause	Covariate	Regression Coefficient	St. Err.	P-value	HR (95% C.I.)	P_{PH}
CHS	all-cause	rLTL	-0.2733	0.0657	3.2E-05	1.31 (1.16, 1.49)	0.02
CHS	all-cause	sex	0.1107	0.0708	0.12	1.12 (0.97, 1.28)	0.24
CHS	CVD	rLTL	-0.1538	0.1108	0.17	1.17 (0.94, 1.45)	0.11
CHS	CVD	sex	0.1510	0.1198	0.21	1.16 (0.92, 1.47)	0.66
CHS	OC	rLTL	-0.3334	0.0949	0.0004	1.40 (1.16, 1.68)	0.12
CHS	OC	sex	0.0283	0.1031	0.78	1.03 (0.84, 1.26)	0.60
CHS	cancer	rLTL	-0.3585	0.1590	0.02	1.43 (1.05, 1.95)	0.59
CHS	cancer	sex	0.2548	0.1695	0.13	1.29 (0.93, 1.80)	0.23
FHS	all-cause	rLTL	-0.3522	0.1097	0.001	1.42 (1.15, 1.76)	0.82
FHS	all-cause	sex	0.5143	0.1110	3.6E-06	1.67 (1.35, 2.08)	0.26
FHS	CVD	rLTL	-0.3899	0.2302	0.09	1.48 (0.94, 2.32)	0.31
FHS	CVD	sex	1.0225	0.2465	3.3E-05	2.78 (1.72, 4.51)	0.79
FHS	OC	rLTL	-0.4819	0.1725	0.005	1.62 (1.15, 2.27)	0.71
FHS	OC	sex	0.2652	0.1685	0.12	1.30 (0.94, 1.81)	0.77
FHS	cancer	rLTL	-0.1832	0.1813	0.31	1.20 (0.84, 1.71)	0.87
FHS	cancer	sex	0.5009	0.1895	0.008	1.65 (1.14, 2.39)	0.31
WHI	all-cause	rLTL	-0.2840	0.0983	0.004	1.33 (1.10, 1.61)	0.57
WHI	CVD	rLTL	-0.3525	0.1739	0.04	1.42 (1.01, 2.00)	0.38
WHI	OC	rLTL	-0.7228	0.1735	3.1E-05	2.06 (1.47, 2.89)	0.41
WHI	cancer	rLTL	0.1953	0.1652	0.24	0.82 (0.60, 1.14)	0.27

Notes: See Notes to eTable 2 regarding covariates, abbreviations and names of columns.

© 2020 Arbeev KG et al. *JAMA Network Open.*

eTable 5. Results of Analyses of All-Cause and Cause-Specific Mortality in Separate Samples (CHS, FHS, WHI) Using the Model With Stratified Baseline Hazards for Age Groups

Study	Cause	Covariate	Regression Coefficient	St. Err.	P-value	HR (95% C.I.)	P_{PH}
CHS	all-cause	a_0^*	0.1293	0.0122	2.8E-26	1.14 (1.11, 1.17)	0.04
CHS	all-cause	rLTL	-0.2673	0.0656	4.7E-05	1.31 (1.15, 1.49)	0.04
CHS	all-cause	sex	0.0867	0.0706	0.22	1.09 (0.95, 1.25)	0.27
CHS	CVD	a_0^*	0.1249	0.0205	1.0E-09	1.13 (1.09, 1.18)	0.26
CHS	CVD	rLTL	-0.1485	0.1104	0.18	1.16 (0.93, 1.44)	0.17
CHS	CVD	sex	0.1236	0.1197	0.30	1.13 (0.89, 1.43)	0.59
CHS	OC	a_0^*	0.1576	0.0175	2.6E-19	1.17 (1.13, 1.21)	0.50
CHS	OC	rLTL	-0.3259	0.0951	0.0006	1.39 (1.15, 1.67)	0.13
CHS	OC	sex	0.0119	0.1025	0.91	1.01 (0.83, 1.24)	0.73
CHS	cancer	a_0^*	0.0565	0.0304	0.06	1.06 (1.00, 1.12)	0.004
CHS	cancer	rLTL	-0.3423	0.1597	0.03	1.41 (1.03, 1.93)	0.74
CHS	cancer	sex	0.2180	0.1695	0.20	1.24 (0.89, 1.73)	0.28
FHS	all-cause	a_0^*	0.0975	0.0120	5.7E-16	1.10 (1.08, 1.13)	0.13
FHS	all-cause	rLTL	-0.3466	0.1099	0.002	1.41 (1.14, 1.75)	0.77
FHS	all-cause	sex	0.4998	0.1109	6.6E-06	1.65 (1.33, 2.05)	0.34
FHS	CVD	a_0^*	0.1262	0.0277	5.3E-06	1.13 (1.07, 1.20)	0.14
FHS	CVD	rLTL	-0.3952	0.2305	0.09	1.48 (0.94, 2.33)	0.26
FHS	CVD	sex	0.9621	0.2446	8.4E-05	2.62 (1.62, 4.23)	0.90
FHS	OC	a_0^*	0.1447	0.0204	1.5E-12	1.16 (1.11, 1.20)	0.54
FHS	OC	rLTL	-0.4799	0.1737	0.006	1.62 (1.15, 2.27)	0.70
FHS	OC	sex	0.2861	0.1688	0.09	1.33 (0.96, 1.85)	0.93
FHS	cancer	a_0^*	0.0437	0.0183	0.02	1.04 (1.01, 1.08)	0.45
FHS	cancer	rLTL	-0.1593	0.1810	0.38	1.17 (0.82, 1.67)	0.87
FHS	cancer	sex	0.4879	0.1890	0.010	1.63 (1.12, 2.36)	0.33
WHI	all-cause	a_0^*	0.0635	0.0143	9.3E-06	1.07 (1.04, 1.10)	0.002
WHI	all-cause	rLTL	-0.2836	0.0981	0.004	1.33 (1.10, 1.61)	0.55
WHI	CVD	a_0^*	0.0814	0.0267	0.002	1.08 (1.03, 1.14)	0.27
WHI	CVD	rLTL	-0.3564	0.1727	0.04	1.43 (1.02, 2.00)	0.36
WHI	OC	a_0^*	0.0909	0.0257	0.0004	1.10 (1.04, 1.15)	0.07
WHI	OC	rLTL	-0.7225	0.1748	3.6E-05	2.06 (1.46, 2.90)	0.46
WHI	cancer	a_0^*	0.0271	0.0226	0.23	1.03 (0.98, 1.07)	0.26
WHI	cancer	rLTL	0.1920	0.1643	0.24	0.83 (0.60, 1.14)	0.27

Notes: See Notes to eTable 3 regarding covariates, abbreviations and names of columns.

© 2020 Arbeev KG et al. *JAMA Network Open.*

eTable 6. Hazard Ratios for All-Cause Mortality for Different Ages and Residual Leukocyte Telomere Length (rLTL) Computed From the Cox Model With Splines for Age

Age\ rLTL	-1.5	-1.4	-1.3	-1.2	-1.1	-1.0	-0.9	-0.8
50	1.5 (1.3, 1.8)	1.5 (1.3, 1.7)	1.5 (1.3, 1.7)	1.4 (1.3, 1.6)	1.4 (1.2, 1.5)	1.3 (1.2, 1.5)	1.3 (1.2, 1.4)	1.3 (1.2, 1.4)
55	2.6 (2.2, 3.1)	2.5 (2.2, 3.0)	2.5 (2.1, 2.8)	2.4 (2.1, 2.7)	2.3 (2.0, 2.6)	2.2 (2.0, 2.6)	2.2 (1.9, 2.5)	2.1 (1.9, 2.4)
60	4.4 (3.6, 5.5)	4.3 (3.5, 5.3)	4.2 (3.4, 5.1)	4.1 (3.3, 4.9)	3.9 (3.3, 4.8)	3.8 (3.2, 4.6)	3.7 (3.1, 4.5)	3.6 (3.0, 4.3)
65	7.8 (6.0, 10.1)	7.6 (5.9, 9.8)	7.4 (5.7, 9.5)	7.1 (5.6, 9.2)	6.9 (5.4, 8.9)	6.7 (5.3, 8.6)	6.6 (5.2, 8.3)	6.4 (5.0, 8.0)
70	14.6 (10.8, 19.7)	14.2 (10.5, 19.0)	13.8 (10.3, 18.4)	13.4 (10.0, 17.8)	13.0 (9.8, 17.2)	12.6 (9.5, 16.7)	12.2 (9.3, 16.1)	11.9 (9.1, 15.6)
75	29.6 (21.6, 40.4)	28.7 (21.1, 39.1)	27.9 (20.6, 37.8)	27.1 (20.1, 36.6)	26.3 (19.6, 35.4)	25.6 (19.1, 34.2)	24.8 (18.6, 33.2)	24.1 (18.1, 32.1)
80	66.7 (49.2, 90.5)	64.8 (48.0, 87.5)	63.0 (46.8, 84.6)	61.2 (45.7, 81.9)	59.4 (44.6, 79.2)	57.7 (43.5, 76.7)	56.1 (42.4, 74.2)	54.5 (41.3, 71.9)
85	151.6 (112.4, 204.5)	147.2 (109.7, 197.7)	143.0 (107.0, 191.1)	138.9 (104.4, 184.8)	135.0 (101.9, 178.8)	131.1 (99.4, 173.0)	127.4 (96.9, 167.5)	123.7 (94.4, 162.1)
90	198.0 (129.7, 302.3)	192.4 (126.4, 292.7)	186.9 (123.2, 283.5)	181.5 (120.0, 274.6)	176.3 (116.8, 266.1)	171.3 (113.7, 257.9)	166.4 (110.7, 250.0)	161.6 (107.8, 242.4)

Age\ rLTL	-0.7	-0.6	-0.5	-0.4	-0.3	-0.2	-0.1	0
50	1.2 (1.1, 1.3)	1.2 (1.1, 1.3)	1.2 (1.1, 1.2)	1.1 (1.1, 1.2)	1.1 (1.1, 1.1)	1.1 (1.0, 1.1)	1.0 (1.0, 1.0)	1.0 (1.0, 1.0)
55	2.1 (1.9, 2.3)	2.0 (1.8, 2.2)	1.9 (1.8, 2.1)	1.9 (1.7, 2.1)	1.8 (1.7, 2.0)	1.8 (1.6, 1.9)	1.7 (1.6, 1.9)	1.7 (1.6, 1.8)
60	3.5 (3.0, 4.2)	3.4 (2.9, 4.0)	3.3 (2.8, 3.9)	3.2 (2.7, 3.8)	3.1 (2.7, 3.7)	3.0 (2.6, 3.6)	2.9 (2.5, 3.5)	2.9 (2.4, 3.4)
65	6.2 (4.9, 7.8)	6.0 (4.8, 7.5)	5.8 (4.7, 7.3)	5.7 (4.5, 7.1)	5.5 (4.4, 6.9)	5.3 (4.3, 6.7)	5.2 (4.2, 6.5)	5.0 (4.1, 6.3)
70	11.6 (8.8, 15.1)	11.2 (8.6, 14.7)	10.9 (8.4, 14.2)	10.6 (8.1, 13.8)	10.3 (7.9, 13.4)	10.0 (7.7, 13.0)	9.7 (7.5, 12.6)	9.4 (7.3, 12.2)
75	23.4 (17.7, 31.1)	22.8 (17.2, 30.2)	22.1 (16.7, 29.2)	21.5 (16.3, 28.4)	20.9 (15.8, 27.5)	20.3 (15.4, 26.7)	19.7 (15.0, 25.9)	19.1 (14.6, 25.2)
80	52.9 (40.2, 69.6)	51.4 (39.2, 67.5)	49.9 (38.1, 65.4)	48.5 (37.1, 63.4)	47.1 (36.1, 61.5)	45.8 (35.1, 59.6)	44.5 (34.1, 57.9)	43.2 (33.2, 56.2)
85	120.2 (92.0, 157.0)	116.7 (89.6, 152.1)	113.4 (87.2, 147.4)	110.2 (84.9, 142.9)	107.0 (82.6, 138.6)	104.0 (80.4, 134.4)	101.0 (78.2, 130.5)	98.1 (76.0, 126.7)

© 2020 Arbeev KG et al. *JAMA Network Open.*

**90**	157.0 (104.9, 235.1)	152.5 (102.0, 228.0)	148.2 (99.2, 221.3)	143.9 (96.5, 214.7)	139.8 (93.8, 208.4)	135.8 (91.1, 202.4)	131.9 (88.6, 196.5)	128.2 (86.0, 190.9)

**Age\ rLTL**	**0.1**	**0.2**	**0.3**	**0.4**	**0.5**	**0.6**	**0.7**	**0.8**
**50**	1.0 (1.0, 1.0)	0.9 (0.9, 1.0)	0.9 (0.9, 0.9)	0.9 (0.9, 0.9)	0.9 (0.8, 0.9)	0.8 (0.8, 0.9)	0.8 (0.8, 0.9)	0.8 (0.7, 0.9)
**55**	1.6 (1.5, 1.8)	1.6 (1.5, 1.7)	1.5 (1.4, 1.7)	1.5 (1.4, 1.6)	1.5 (1.3, 1.6)	1.4 (1.3, 1.6)	1.4 (1.2, 1.5)	1.3 (1.2, 1.5)
**60**	2.8 (2.4, 3.3)	2.7 (2.3, 3.2)	2.6 (2.2, 3.1)	2.6 (2.2, 3.0)	2.5 (2.1, 2.9)	2.4 (2.0, 2.8)	2.3 (2.0, 2.8)	2.3 (1.9, 2.7)
**65**	4.9 (3.9, 6.1)	4.8 (3.8, 5.9)	4.6 (3.7, 5.8)	4.5 (3.6, 5.6)	4.4 (3.5, 5.5)	4.2 (3.4, 5.3)	4.1 (3.3, 5.2)	4.0 (3.2, 5.0)
**70**	9.2 (7.1, 11.9)	8.9 (6.9, 11.6)	8.6 (6.7, 11.2)	8.4 (6.5, 10.9)	8.2 (6.3, 10.6)	7.9 (6.1, 10.3)	7.7 (5.9, 10.1)	7.5 (5.7, 9.8)
**75**	18.6 (14.1, 24.4)	18.1 (13.7, 23.7)	17.5 (13.3, 23.1)	17.0 (12.9, 22.4)	16.6 (12.6, 21.8)	16.1 (12.2, 21.2)	15.6 (11.8, 20.7)	15.2 (11.4, 20.1)
**80**	42.0 (32.2, 54.6)	40.8 (31.3, 53.0)	39.6 (30.4, 51.5)	38.5 (29.5, 50.1)	37.4 (28.6, 48.7)	36.3 (27.8, 47.4)	35.3 (26.9, 46.2)	34.2 (26.1, 44.9)
**85**	95.3 (73.8, 123.0)	92.6 (71.7, 119.5)	89.9 (69.6, 116.1)	87.4 (67.6, 112.9)	84.9 (65.6, 109.8)	82.4 (63.6, 106.8)	80.1 (61.7, 104.0)	77.8 (59.8, 101.2)
**90**	124.5 (83.6, 185.4)	120.9 (81.1, 180.2)	117.5 (78.8, 175.2)	114.1 (76.5, 170.3)	110.8 (74.2, 165.6)	107.7 (72.0, 161.1)	104.6 (69.8, 156.7)	101.6 (67.7, 152.5)

**Age\ rLTL**	**0.9**	**1**	**1.1**	**1.2**	**1.3**	**1.4**	**1.5**	
**50**	0.8 (0.7, 0.8)	0.7 (0.7, 0.8)	0.7 (0.7, 0.8)	0.7 (0.6, 0.8)	0.7 (0.6, 0.8)	0.7 (0.6, 0.8)	0.6 (0.6, 0.7)	
**55**	1.3 (1.2, 1.5)	1.3 (1.1, 1.4)	1.2 (1.1, 1.4)	1.2 (1.0, 1.4)	1.2 (1.0, 1.3)	1.1 (1.0, 1.3)	1.1 (0.9, 1.3)	
**60**	2.2 (1.8, 2.6)	2.1 (1.8, 2.6)	2.1 (1.7, 2.5)	2.0 (1.7, 2.5)	2.0 (1.6, 2.4)	1.9 (1.6, 2.3)	1.9 (1.5, 2.3)	
**65**	3.9 (3.1, 4.9)	3.8 (3.0, 4.8)	3.7 (2.9, 4.7)	3.6 (2.8, 4.6)	3.5 (2.7, 4.5)	3.4 (2.6, 4.3)	3.3 (2.5, 4.2)	
**70**	7.3 (5.5, 9.5)	7.1 (5.4, 9.3)	6.9 (5.2, 9.1)	6.7 (5.0, 8.8)	6.5 (4.9, 8.6)	6.3 (4.7, 8.4)	6.1 (4.5, 8.2)	
**75**	14.7 (11.1, 19.6)	14.3 (10.7, 19.1)	13.9 (10.4, 18.6)	13.5 (10.1, 18.1)	13.1 (9.7, 17.7)	12.7 (9.4, 17.3)	12.4 (9.1, 16.8)	
**80**	33.3 (25.3, 43.8)	32.3 (24.5, 42.6)	31.4 (23.7, 41.5)	30.5 (23.0, 40.5)	29.6 (22.2, 39.5)	28.8 (21.5, 38.5)	28.0 (20.8, 37.6)	
**85**	75.6 (57.9, 98.6)	73.4 (56.1, 96.0)	71.3 (54.3, 93.6)	69.3 (52.6, 91.2)	67.3 (50.9, 88.9)	65.4 (49.3, 86.7)	63.5 (47.6, 84.6)	

© 2020 Arbeev KG et al. *JAMA Network Open.*

**90**	98.7<br>(65.6, 148.5)	95.9<br>(63.6, 144.5)	93.1<br>(61.6, 140.7)	90.5<br>(59.7, 137.1)	87.9<br>(57.9, 133.5)	85.4<br>(56.0, 130.1)	82.9<br>(54.3, 126.8)	

eTable 7. Hazard Ratios for Cardiovascular Disease Mortality for Different Ages and Residual Leukocyte Telomere Length (rLTL) Computed From the Cox Model With Splines for Age

Age\ rLTL	-1.5	-1.4	-1.3	-1.2	-1.1	-1.0	-0.9	-0.8
**50**	1.4 (1.1, 1.9)	1.4 (1.1, 1.8)	1.4 (1.1, 1.7)	1.3 (1.1, 1.6)	1.3 (1.1, 1.6)	1.3 (1.1, 1.5)	1.2 (1.1, 1.5)	1.2 (1.1, 1.4)
**55**	2.9 (2.1, 3.9)	2.8 (2.1, 3.7)	2.7 (2.1, 3.6)	2.7 (2.0, 3.5)	2.6 (2.0, 3.3)	2.5 (2.0, 3.2)	2.5 (2.0, 3.1)	2.4 (1.9, 3.0)
**60**	5.7 (3.8, 8.7)	5.6 (3.7, 8.4)	5.5 (3.7, 8.1)	5.3 (3.6, 7.8)	5.2 (3.6, 7.6)	5.1 (3.5, 7.3)	4.9 (3.5, 7.1)	4.8 (3.4, 6.9)
**65**	11.8 (7.0, 19.9)	11.5 (6.9, 19.2)	11.2 (6.7, 18.6)	10.9 (6.6, 18.0)	10.7 (6.5, 17.5)	10.4 (6.4, 17.0)	10.2 (6.3, 16.5)	9.9 (6.2, 16.0)
**70**	25.2 (13.8, 46.2)	24.6 (13.5, 44.7)	24.0 (13.3, 43.4)	23.4 (13.1, 42.1)	22.9 (12.8, 40.8)	22.3 (12.6, 39.6)	21.8 (12.3, 38.4)	21.3 (12.1, 37.4)
**75**	57.5 (30.4, 108.8)	56.1 (29.9, 105.4)	54.8 (29.3, 102.2)	53.4 (28.8, 99.1)	52.1 (28.3, 96.2)	50.9 (27.7, 93.4)	49.7 (27.2, 90.7)	48.5 (26.6, 88.1)
**80**	141.8 (76.1, 264.2)	138.4 (74.8, 255.9)	135.0 (73.5, 248.0)	131.8 (72.2, 240.5)	128.6 (70.9, 233.2)	125.5 (69.6, 226.3)	122.4 (68.2, 219.7)	119.5 (66.9, 213.4)
**85**	333.1 (183.6, 604.1)	325.0 (180.6, 584.9)	317.2 (177.6, 566.6)	309.5 (174.5, 549.0)	302.0 (171.4, 532.3)	294.7 (168.3, 516.2)	287.6 (165.1, 500.9)	280.7 (162.0, 486.3)
**90**	383.1 (171.5, 855.9)	373.9 (168.2, 831.0)	364.8 (164.9, 807.0)	356.0 (161.7, 784.0)	347.4 (158.4, 761.9)	339.0 (155.2, 740.7)	330.9 (152.0, 720.4)	322.9 (148.7, 700.8)

Age\ rLTL	-0.7	-0.6	-0.5	-0.4	-0.3	-0.2	-0.1	0
**50**	1.2 (1.1, 1.3)	1.2 (1.0, 1.3)	1.1 (1.0, 1.2)	1.1 (1.0, 1.2)	1.1 (1.0, 1.1)	1.1 (1.0, 1.1)	1.0 (1.0, 1.0)	1.0 (1.0, 1.0)
**55**	2.4 (1.9, 2.9)	2.3 (1.9, 2.8)	2.2 (1.9, 2.7)	2.2 (1.8, 2.6)	2.1 (1.8, 2.5)	2.1 (1.8, 2.5)	2.0 (1.7, 2.4)	2.0 (1.7, 2.3)
**60**	4.7 (3.3, 6.7)	4.6 (3.3, 6.5)	4.5 (3.2, 6.3)	4.4 (3.1, 6.1)	4.3 (3.1, 5.9)	4.2 (3.0, 5.8)	4.1 (2.9, 5.6)	4.0 (2.9, 5.5)
**65**	9.7 (6.0, 15.5)	9.4 (5.9, 15.1)	9.2 (5.8, 14.7)	9.0 (5.7, 14.3)	8.8 (5.5, 13.9)	8.6 (5.4, 13.5)	8.4 (5.3, 13.2)	8.2 (5.2, 12.9)
**70**	20.7 (11.9, 36.3)	20.2 (11.6, 35.3)	19.8 (11.4, 34.3)	19.3 (11.1, 33.4)	18.8 (10.9, 32.6)	18.4 (10.6, 31.7)	17.9 (10.4, 30.9)	17.5 (10.1, 30.2)
**75**	47.3 (26.1, 85.6)	46.1 (25.6, 83.3)	45.0 (25.0, 81.0)	43.9 (24.5, 78.9)	42.9 (23.9, 76.8)	41.8 (23.4, 74.9)	40.8 (22.8, 73.0)	39.8 (22.3, 71.2)
**80**	116.6 (65.6, 207.3)	113.8 (64.2, 201.6)	111.0 (62.9, 196.0)	108.4 (61.5, 190.8)	105.7 (60.2, 185.7)	103.2 (58.8, 180.9)	100.7 (57.5, 176.3)	98.3 (56.2, 171.9)
**85**	273.9 (158.8, 472.3)	267.3 (155.6, 459.0)	260.8 (152.4, 446.3)	254.5 (149.2, 434.1)	248.4 (146.0, 422.5)	242.4 (142.8, 411.5)	236.5 (139.5, 400.9)	230.8 (136.3, 390.9)

© 2020 Arbeev KG et al. *JAMA Network Open.*

**90**	315.1 (145.5, 682.1)	307.5 (142.3, 664.1)	300.0 (139.2, 646.8)	292.8 (136.0, 630.2)	285.7 (132.9, 614.2)	278.8 (129.8, 598.9)	272.1 (126.7, 584.2)	265.5 (123.7, 570.1)

Age\ rLTL	0.1	0.2	0.3	0.4	0.5	0.6	0.7	0.8
**50**	1.0 (1.0, 1.0)	1.0 (0.9, 1.0)	0.9 (0.9, 1.0)	0.9 (0.8, 1.0)	0.9 (0.8, 1.0)	0.9 (0.8, 1.0)	0.8 (0.7, 1.0)	0.8 (0.7, 0.9)
**55**	1.9 (1.6, 2.3)	1.9 (1.6, 2.2)	1.8 (1.5, 2.2)	1.8 (1.5, 2.2)	1.8 (1.5, 2.1)	1.7 (1.4, 2.1)	1.7 (1.4, 2.1)	1.6 (1.3, 2.0)
**60**	3.9 (2.8, 5.4)	3.8 (2.7, 5.2)	3.7 (2.7, 5.1)	3.6 (2.6, 5.0)	3.5 (2.5, 4.9)	3.4 (2.4, 4.8)	3.3 (2.4, 4.7)	3.3 (2.3, 4.6)
**65**	8.0 (5.0, 12.6)	7.8 (4.9, 12.3)	7.6 (4.8, 12.0)	7.4 (4.7, 11.7)	7.2 (4.5, 11.5)	7.0 (4.4, 11.2)	6.9 (4.3, 11.0)	6.7 (4.2, 10.8)
**70**	17.1 (9.9, 29.5)	16.6 (9.6, 28.8)	16.2 (9.4, 28.1)	15.9 (9.1, 27.5)	15.5 (8.9, 26.9)	15.1 (8.7, 26.3)	14.7 (8.4, 25.7)	14.4 (8.2, 25.2)
**75**	38.9 (21.8, 69.5)	37.9 (21.2, 67.8)	37.0 (20.7, 66.2)	36.1 (20.2, 64.7)	35.3 (19.6, 63.3)	34.4 (19.1, 61.9)	33.6 (18.6, 60.6)	32.8 (18.1, 59.4)
**80**	95.9 (54.8, 167.7)	93.6 (53.5, 163.7)	91.3 (52.1, 159.9)	89.1 (50.8, 156.3)	87.0 (49.5, 152.8)	84.9 (48.2, 149.4)	82.8 (46.9, 146.2)	80.8 (45.6, 143.2)
**85**	225.2 (133.1, 381.3)	219.8 (129.8, 372.1)	214.5 (126.6, 363.4)	209.3 (123.4, 355.1)	204.3 (120.2, 347.1)	199.3 (117.0, 339.5)	194.5 (113.9, 332.3)	189.8 (110.7, 325.4)
**90**	259.1 (120.6, 556.5)	252.8 (117.6, 543.5)	246.7 (114.7, 531.0)	240.8 (111.7, 518.9)	235.0 (108.8, 507.4)	229.3 (105.9, 496.2)	223.7 (103.1, 485.5)	218.3 (100.3, 475.2)

Age\ rLTL	0.9	1	1.1	1.2	1.3	1.4	1.5	
**50**	0.8 (0.7, 0.9)	0.8 (0.7, 0.9)	0.8 (0.6, 0.9)	0.7 (0.6, 0.9)	0.7 (0.6, 0.9)	0.7 (0.6, 0.9)	0.7 (0.5, 0.9)	
**55**	1.6 (1.3, 2.0)	1.6 (1.2, 2.0)	1.5 (1.2, 2.0)	1.5 (1.1, 1.9)	1.4 (1.1, 1.9)	1.4 (1.1, 1.9)	1.4 (1.0, 1.9)	
**60**	3.2 (2.2, 4.6)	3.1 (2.2, 4.5)	3.0 (2.1, 4.4)	3.0 (2.0, 4.4)	2.9 (1.9, 4.3)	2.8 (1.9, 4.2)	2.8 (1.8, 4.2)	
**65**	6.5 (4.0, 10.6)	6.4 (3.9, 10.4)	6.2 (3.8, 10.2)	6.1 (3.7, 10.0)	5.9 (3.6, 9.9)	5.8 (3.5, 9.7)	5.6 (3.3, 9.5)	
**70**	14.0 (8.0, 24.7)	13.7 (7.7, 24.2)	13.4 (7.5, 23.8)	13.0 (7.3, 23.3)	12.7 (7.1, 22.9)	12.4 (6.8, 22.5)	12.1 (6.6, 22.1)	
**75**	32.0 (17.6, 58.2)	31.2 (17.1, 57.0)	30.4 (16.6, 55.9)	29.7 (16.1, 54.8)	29.0 (15.6, 53.8)	28.3 (15.1, 52.9)	27.6 (14.7, 51.9)	
**80**	78.9 (44.3, 140.3)	76.9 (43.1, 137.5)	75.1 (41.8, 134.8)	73.3 (40.6, 132.3)	71.5 (39.4, 129.8)	69.8 (38.2, 127.5)	68.1 (37.0, 125.2)	
**85**	185.2 (107.6, 318.8)	180.8 (104.6, 312.5)	176.4 (101.5, 306.4)	172.1 (98.5, 300.7)	168.0 (95.6, 295.1)	163.9 (92.7, 289.8)	160.0 (89.8, 284.8)	

© 2020 Arbeev KG et al. *JAMA Network Open.*

**90**	213.1<br>(97.6, 465.3)	207.9<br>(94.9, 455.8)	202.9<br>(92.2, 446.6)	198.0<br>(89.6, 437.7)	193.2<br>(87.0, 429.2)	188.6<br>(84.4, 421.0)	184.0<br>(82.0, 413.1)	

© 2020 Arbeev KG et al. *JAMA Network Open.*

eTable 8. Hazard Ratios for Other Causes Mortality for Different Ages and Residual Leukocyte Telomere Length (rLTL) Computed From the Cox Model With Splines for Age

Age\ rLTL	-1.5	-1.4	-1.3	-1.2	-1.1	-1.0	-0.9	-0.8
**50**	1.9 (1.5, 2.4)	1.8 (1.5, 2.2)	1.7 (1.4, 2.1)	1.7 (1.4, 2.0)	1.6 (1.4, 1.9)	1.5 (1.3, 1.8)	1.5 (1.3, 1.7)	1.4 (1.2, 1.6)
**55**	4.1 (3.1, 5.3)	3.9 (3.0, 5.0)	3.7 (2.9, 4.7)	3.6 (2.8, 4.5)	3.4 (2.8, 4.3)	3.3 (2.7, 4.0)	3.1 (2.6, 3.8)	3.0 (2.5, 3.6)
**60**	8.7 (6.1, 12.5)	8.4 (5.9, 11.9)	8.0 (5.7, 11.3)	7.7 (5.5, 10.7)	7.4 (5.3, 10.2)	7.1 (5.1, 9.7)	6.8 (4.9, 9.2)	6.5 (4.8, 8.8)
**65**	19.0 (12.1, 30.0)	18.2 (11.7, 28.6)	17.5 (11.2, 27.2)	16.8 (10.8, 25.9)	16.1 (10.4, 24.7)	15.4 (10.1, 23.5)	14.7 (9.7, 22.4)	14.1 (9.3, 21.4)
**70**	42.6 (25.2, 72.0)	40.8 (24.3, 68.6)	39.1 (23.4, 65.3)	37.5 (22.6, 62.3)	35.9 (21.7, 59.4)	34.4 (20.9, 56.6)	33.0 (20.1, 54.0)	31.6 (19.4, 51.6)
**75**	98.5 (56.7, 171.3)	94.4 (54.6, 163.2)	90.5 (52.6, 155.5)	86.7 (50.7, 148.3)	83.1 (48.8, 141.4)	79.6 (47.0, 134.9)	76.3 (45.2, 128.7)	73.1 (43.5, 122.9)
**80**	238.3 (139.0, 408.8)	228.4 (134.0, 389.3)	218.9 (129.2, 370.8)	209.8 (124.5, 353.4)	201.0 (120.0, 336.9)	192.6 (115.5, 321.3)	184.6 (111.2, 306.5)	176.9 (107.0, 292.5)
**85**	571.9 (341.2, 958.5)	548.1 (329.2, 912.4)	525.3 (317.5, 868.9)	503.4 (306.1, 827.6)	482.4 (295.0, 788.7)	462.3 (284.2, 751.8)	443.0 (273.7, 717.0)	424.5 (263.5, 684.0)
**90**	1025.7 (544.7, 1931.3)	982.9 (524.7, 1841.5)	942.0 (505.2, 1756.5)	902.7 (486.3, 1675.9)	865.1 (467.9, 1599.5)	829.0 (450.1, 1527.1)	794.5 (432.8, 1458.4)	761.4 (416.0, 1393.4)

Age\ rLTL	-0.7	-0.6	-0.5	-0.4	-0.3	-0.2	-0.1	0
**50**	1.3 (1.2, 1.5)	1.3 (1.2, 1.4)	1.2 (1.1, 1.3)	1.2 (1.1, 1.3)	1.1 (1.1, 1.2)	1.1 (1.1, 1.1)	1.0 (1.0, 1.1)	1.0 (1.0, 1.0)
**55**	2.9 (2.4, 3.4)	2.8 (2.3, 3.3)	2.6 (2.3, 3.1)	2.5 (2.2, 3.0)	2.4 (2.1, 2.8)	2.3 (2.0, 2.7)	2.2 (1.9, 2.6)	2.1 (1.9, 2.5)
**60**	6.2 (4.6, 8.4)	5.9 (4.4, 8.0)	5.7 (4.3, 7.6)	5.5 (4.1, 7.3)	5.2 (3.9, 7.0)	5.0 (3.8, 6.7)	4.8 (3.6, 6.4)	4.6 (3.5, 6.1)
**65**	13.5 (9.0, 20.4)	13.0 (8.6, 19.5)	12.4 (8.3, 18.6)	11.9 (8.0, 17.8)	11.4 (7.7, 17.0)	10.9 (7.4, 16.3)	10.5 (7.1, 15.6)	10.1 (6.8, 15.0)
**70**	30.3 (18.6, 49.2)	29.0 (17.9, 47.1)	27.8 (17.2, 45.0)	26.7 (16.5, 43.0)	25.5 (15.9, 41.1)	24.5 (15.2, 39.4)	23.5 (14.6, 37.7)	22.5 (14.0, 36.1)
**75**	70.1 (41.9, 117.4)	67.2 (40.2, 112.1)	64.4 (38.7, 107.2)	61.7 (37.1, 102.5)	59.1 (35.6, 98.1)	56.7 (34.2, 93.8)	54.3 (32.8, 89.8)	52.0 (31.5, 86.1)
**80**	169.6 (102.9, 279.3)	162.5 (99.0, 266.7)	155.7 (95.1, 254.9)	149.2 (91.4, 243.7)	143.0 (87.8, 233.0)	137.0 (84.2, 223.0)	131.3 (80.8, 213.4)	125.9 (77.5, 204.4)
**85**	406.9 (253.5, 652.9)	389.9 (243.9, 623.4)	373.6 (234.5, 595.5)	358.1 (225.3, 569.1)	343.2 (216.4, 544.1)	328.9 (207.8, 520.5)	315.1 (199.4, 498.1)	302.0 (191.2, 476.9)

© 2020 Arbeev KG et al. *JAMA Network Open.*

90	729.6 (399.8, 1331.6)	699.2 (384.1, 1273.1)	670.1 (368.8, 1217.6)	642.2 (354.0, 1164.9)	615.4 (339.7, 1114.9)	589.8 (325.9, 1067.4)	565.2 (312.5, 1022.3)	541.6 (299.5, 979.5)

Age\ rLTL	0.1	0.2	0.3	0.4	0.5	0.6	0.7	0.8
50	1.0 (0.9, 1.0)	0.9 (0.9, 0.9)	0.9 (0.8, 0.9)	0.8 (0.8, 0.9)	0.8 (0.8, 0.9)	0.8 (0.7, 0.8)	0.7 (0.7, 0.8)	0.7 (0.6, 0.8)
55	2.1 (1.8, 2.4)	2.0 (1.7, 2.3)	1.9 (1.6, 2.2)	1.8 (1.5, 2.1)	1.7 (1.5, 2.0)	1.7 (1.4, 2.0)	1.6 (1.3, 1.9)	1.5 (1.3, 1.8)
60	4.4 (3.3, 5.9)	4.2 (3.2, 5.6)	4.1 (3.0, 5.4)	3.9 (2.9, 5.2)	3.7 (2.8, 5.0)	3.6 (2.7, 4.8)	3.4 (2.5, 4.6)	3.3 (2.4, 4.4)
65	9.6 (6.5, 14.3)	9.2 (6.2, 13.7)	8.8 (5.9, 13.2)	8.5 (5.7, 12.7)	8.1 (5.4, 12.2)	7.8 (5.2, 11.7)	7.5 (5.0, 11.2)	7.2 (4.7, 10.8)
70	21.5 (13.4, 34.6)	20.6 (12.8, 33.2)	19.8 (12.3, 31.8)	19.0 (11.8, 30.6)	18.2 (11.3, 29.3)	17.4 (10.8, 28.2)	16.7 (10.3, 27.1)	16.0 (9.8, 26.0)
75	49.9 (30.1, 82.5)	47.8 (28.9, 79.1)	45.8 (27.7, 75.8)	43.9 (26.5, 72.8)	42.1 (25.3, 69.8)	40.3 (24.2, 67.1)	38.6 (23.1, 64.4)	37.0 (22.1, 61.9)
80	120.6 (74.3, 195.8)	115.6 (71.2, 187.7)	110.8 (68.2, 180.0)	106.2 (65.3, 172.7)	101.7 (62.4, 165.7)	97.5 (59.7, 159.1)	93.4 (57.1, 152.9)	89.5 (54.6, 146.9)
85	289.4 (183.4, 456.9)	277.4 (175.7, 437.8)	265.8 (168.3, 419.8)	254.7 (161.1, 402.7)	244.1 (154.2, 386.5)	233.9 (147.5, 371.1)	224.2 (141.0, 356.5)	214.8 (134.7, 342.6)
90	519.1 (287.0, 938.8)	497.4 (274.9, 900.1)	476.7 (263.2, 863.4)	456.8 (251.9, 828.4)	437.8 (241.0, 795.2)	419.5 (230.5, 763.5)	402.1 (220.4, 733.4)	385.3 (210.7, 704.7)

Age\ rLTL	0.9	1	1.1	1.2	1.3	1.4	1.5	
50	0.7 (0.6, 0.8)	0.7 (0.6, 0.8)	0.6 (0.5, 0.7)	0.6 (0.5, 0.7)	0.6 (0.5, 0.7)	0.6 (0.4, 0.7)	0.5 (0.4, 0.7)	
55	1.5 (1.2, 1.8)	1.4 (1.1, 1.7)	1.3 (1.1, 1.7)	1.3 (1.0, 1.6)	1.2 (1.0, 1.6)	1.2 (0.9, 1.5)	1.1 (0.9, 1.5)	
60	3.1 (2.3, 4.3)	3.0 (2.2, 4.1)	2.9 (2.1, 4.0)	2.8 (2.0, 3.9)	2.6 (1.9, 3.7)	2.5 (1.8, 3.6)	2.4 (1.7, 3.5)	
65	6.9 (4.5, 10.4)	6.6 (4.3, 10.0)	6.3 (4.1, 9.7)	6.0 (3.9, 9.3)	5.8 (3.7, 9.0)	5.5 (3.5, 8.7)	5.3 (3.4, 8.4)	
70	15.3 (9.4, 25.0)	14.7 (9.0, 24.1)	14.1 (8.5, 23.2)	13.5 (8.1, 22.3)	12.9 (7.8, 21.5)	12.4 (7.4, 20.7)	11.9 (7.1, 20.0)	
75	35.5 (21.1, 59.5)	34.0 (20.2, 57.3)	32.6 (19.3, 55.1)	31.2 (18.4, 53.1)	29.9 (17.5, 51.1)	28.7 (16.7, 49.2)	27.5 (15.9, 47.4)	
80	85.8 (52.1, 141.3)	82.2 (49.8, 135.9)	78.8 (47.5, 130.7)	75.5 (45.3, 125.8)	72.4 (43.2, 121.2)	69.4 (41.2, 116.7)	66.5 (39.3, 112.5)	
85	205.9 (128.7, 329.4)	197.3 (122.9, 316.9)	189.1 (117.3, 304.9)	181.2 (111.9, 293.6)	173.7 (106.7, 282.7)	166.4 (101.7, 272.4)	159.5 (96.9, 262.6)	

© 2020 Arbeev KG et al. *JAMA Network Open.*

**90**	369.2 (201.3, 677.4)	353.9 (192.2, 651.3)	339.1 (183.6, 626.5)	325.0 (175.2, 602.8)	311.4 (167.2, 580.2)	298.4 (159.5, 558.6)	286.0 (152.1, 538.0)	

© 2020 Arbeev KG et al. *JAMA Network Open.*

eTable 9. Hazard Ratios for Cancer Mortality for Different Ages and Residual Leukocyte Telomere Length (rLTL) Computed From the Cox Model With Splines for Age

Age\ rLTL	-1.5	-1.4	-1.3	-1.2	-1.1	-1.0	-0.9	-0.8
**50**	1.2 (0.9, 1.6)	1.2 (0.9, 1.5)	1.2 (0.9, 1.5)	1.2 (0.9, 1.4)	1.1 (0.9, 1.4)	1.1 (0.9, 1.4)	1.1 (0.9, 1.3)	1.1 (0.9, 1.3)
**55**	1.5 (1.1, 2.0)	1.5 (1.1, 2.0)	1.4 (1.1, 1.9)	1.4 (1.1, 1.8)	1.4 (1.1, 1.8)	1.4 (1.1, 1.7)	1.4 (1.1, 1.7)	1.4 (1.1, 1.7)
**60**	1.8 (1.3, 2.7)	1.8 (1.3, 2.6)	1.8 (1.3, 2.5)	1.8 (1.3, 2.5)	1.7 (1.3, 2.4)	1.7 (1.3, 2.4)	1.7 (1.3, 2.3)	1.7 (1.3, 2.3)
**65**	2.4 (1.5, 3.7)	2.3 (1.5, 3.6)	2.3 (1.5, 3.5)	2.3 (1.5, 3.5)	2.3 (1.5, 3.4)	2.2 (1.5, 3.3)	2.2 (1.5, 3.2)	2.2 (1.5, 3.2)
**70**	3.3 (2.0, 5.3)	3.2 (2.0, 5.2)	3.2 (2.0, 5.1)	3.2 (2.0, 5.0)	3.1 (2.0, 4.9)	3.1 (2.0, 4.8)	3.0 (2.0, 4.7)	3.0 (2.0, 4.6)
**75**	4.9 (3.0, 8.1)	4.9 (3.0, 8.0)	4.8 (3.0, 7.8)	4.8 (3.0, 7.6)	4.7 (3.0, 7.5)	4.6 (2.9, 7.3)	4.6 (2.9, 7.2)	4.5 (2.9, 7.0)
**80**	8.3 (5.0, 13.8)	8.2 (5.0, 13.5)	8.1 (5.0, 13.2)	8.0 (5.0, 12.9)	7.9 (5.0, 12.6)	7.8 (5.0, 12.4)	7.7 (4.9, 12.1)	7.6 (4.9, 11.9)
**85**	12.1 (7.0, 21.0)	12.0 (7.0, 20.5)	11.8 (7.0, 20.0)	11.7 (7.0, 19.6)	11.6 (6.9, 19.2)	11.4 (6.9, 18.8)	11.3 (6.9, 18.5)	11.1 (6.9, 18.1)
**90**	4.0 (0.6, 28.7)	4.0 (0.6, 28.3)	3.9 (0.6, 27.9)	3.9 (0.6, 27.5)	3.9 (0.5, 27.1)	3.8 (0.5, 26.7)	3.8 (0.5, 26.3)	3.7 (0.5, 26.0)

Age\ rLTL	-0.7	-0.6	-0.5	-0.4	-0.3	-0.2	-0.1	0
**50**	1.1 (1.0, 1.2)	1.1 (1.0, 1.2)	1.1 (1.0, 1.2)	1.0 (1.0, 1.1)	1.0 (1.0, 1.1)	1.0 (1.0, 1.1)	1.0 (1.0, 1.0)	1.0 (1.0, 1.0)
**55**	1.3 (1.1, 1.6)	1.3 (1.1, 1.6)	1.3 (1.1, 1.5)	1.3 (1.1, 1.5)	1.3 (1.1, 1.5)	1.3 (1.1, 1.4)	1.2 (1.1, 1.4)	1.2 (1.1, 1.4)
**60**	1.7 (1.3, 2.2)	1.6 (1.3, 2.2)	1.6 (1.2, 2.1)	1.6 (1.2, 2.1)	1.6 (1.2, 2.1)	1.6 (1.2, 2.0)	1.6 (1.2, 2.0)	1.5 (1.2, 2.0)
**65**	2.2 (1.5, 3.1)	2.1 (1.5, 3.1)	2.1 (1.5, 3.0)	2.1 (1.5, 3.0)	2.1 (1.5, 2.9)	2.0 (1.4, 2.9)	2.0 (1.4, 2.8)	2.0 (1.4, 2.8)
**70**	3.0 (2.0, 4.5)	2.9 (1.9, 4.4)	2.9 (1.9, 4.4)	2.9 (1.9, 4.3)	2.8 (1.9, 4.2)	2.8 (1.9, 4.2)	2.8 (1.9, 4.1)	2.7 (1.8, 4.1)
**75**	4.5 (2.9, 6.9)	4.4 (2.9, 6.8)	4.4 (2.9, 6.7)	4.3 (2.8, 6.5)	4.3 (2.8, 6.4)	4.2 (2.8, 6.4)	4.2 (2.8, 6.3)	4.1 (2.7, 6.2)
**80**	7.6 (4.9, 11.7)	7.5 (4.9, 11.4)	7.4 (4.8, 11.2)	7.3 (4.8, 11.1)	7.2 (4.8, 10.9)	7.1 (4.7, 10.7)	7.0 (4.7, 10.6)	7.0 (4.6, 10.4)
**85**	11.0 (6.8, 17.8)	10.9 (6.8, 17.5)	10.8 (6.7, 17.2)	10.6 (6.7, 16.9)	10.5 (6.6, 16.6)	10.4 (6.6, 16.4)	10.2 (6.5, 16.2)	10.1 (6.4, 16.0)

© 2020 Arbeev KG et al. *JAMA Network Open.*

**90**	3.7<br>(0.5, 25.7)	3.6<br>(0.5, 25.3)	3.6<br>(0.5, 25.0)	3.5<br>(0.5, 24.7)	3.5<br>(0.5, 24.4)	3.5<br>(0.5, 24.1)	3.4<br>(0.5, 23.8)	3.4<br>(0.5, 23.5)

Age\ rLTL	0.1	0.2	0.3	0.4	0.5	0.6	0.7	0.8
**50**	1.0<br>(1.0, 1.0)	1.0<br>(0.9, 1.0)	1.0<br>(0.9, 1.0)	1.0<br>(0.9, 1.0)	0.9<br>(0.9, 1.0)	0.9<br>(0.8, 1.0)	0.9<br>(0.8, 1.0)	0.9<br>(0.8, 1.1)
**55**	1.2<br>(1.1, 1.4)	1.2<br>(1.1, 1.4)	1.2<br>(1.0, 1.4)	1.2<br>(1.0, 1.4)	1.2<br>(1.0, 1.4)	1.1<br>(1.0, 1.4)	1.1<br>(0.9, 1.4)	1.1<br>(0.9, 1.4)
**60**	1.5<br>(1.2, 1.9)	1.5<br>(1.2, 1.9)	1.5<br>(1.1, 1.9)	1.5<br>(1.1, 1.9)	1.4<br>(1.1, 1.9)	1.4<br>(1.1, 1.9)	1.4<br>(1.1, 1.9)	1.4<br>(1.0, 1.9)
**65**	2.0<br>(1.4, 2.8)	1.9<br>(1.4, 2.7)	1.9<br>(1.4, 2.7)	1.9<br>(1.3, 2.7)	1.9<br>(1.3, 2.7)	1.8<br>(1.3, 2.7)	1.8<br>(1.3, 2.6)	1.8<br>(1.2, 2.6)
**70**	2.7<br>(1.8, 4.0)	2.7<br>(1.8, 4.0)	2.6<br>(1.8, 3.9)	2.6<br>(1.7, 3.9)	2.6<br>(1.7, 3.9)	2.5<br>(1.7, 3.8)	2.5<br>(1.7, 3.8)	2.5<br>(1.6, 3.8)
**75**	4.1<br>(2.7, 6.1)	4.0<br>(2.7, 6.0)	4.0<br>(2.6, 6.0)	3.9<br>(2.6, 5.9)	3.9<br>(2.6, 5.9)	3.8<br>(2.5, 5.8)	3.8<br>(2.5, 5.8)	3.7<br>(2.4, 5.8)
**80**	6.9<br>(4.6, 10.3)	6.8<br>(4.5, 10.2)	6.7<br>(4.5, 10.1)	6.6<br>(4.4, 10.0)	6.5<br>(4.3, 9.9)	6.5<br>(4.3, 9.8)	6.4<br>(4.2, 9.7)	6.3<br>(4.1, 9.7)
**85**	10.0<br>(6.4, 15.8)	9.9<br>(6.3, 15.6)	9.8<br>(6.2, 15.4)	9.7<br>(6.1, 15.3)	9.5<br>(6.0, 15.1)	9.4<br>(5.9, 15.0)	9.3<br>(5.8, 14.9)	9.2<br>(5.7, 14.8)
**90**	3.3<br>(0.5, 23.2)	3.3<br>(0.5, 23.0)	3.3<br>(0.5, 22.7)	3.2<br>(0.5, 22.4)	3.2<br>(0.5, 22.2)	3.1<br>(0.4, 22.0)	3.1<br>(0.4, 21.7)	3.1<br>(0.4, 21.5)

Age\ rLTL	0.9	1	1.1	1.2	1.3	1.4	1.5	
**50**	0.9<br>(0.8, 1.1)	0.9<br>(0.7, 1.1)	0.9<br>(0.7, 1.1)	0.9<br>(0.7, 1.1)	0.9<br>(0.7, 1.1)	0.8<br>(0.6, 1.1)	0.8<br>(0.6, 1.1)	
**55**	1.1<br>(0.9, 1.4)	1.1<br>(0.9, 1.4)	1.1<br>(0.8, 1.4)	1.1<br>(0.8, 1.4)	1.1<br>(0.8, 1.4)	1.0<br>(0.8, 1.4)	1.0<br>(0.8, 1.4)	
**60**	1.4<br>(1.0, 1.9)	1.4<br>(1.0, 1.9)	1.3<br>(1.0, 1.9)	1.3<br>(0.9, 1.9)	1.3<br>(0.9, 1.9)	1.3<br>(0.9, 1.9)	1.3<br>(0.9, 1.9)	
**65**	1.8<br>(1.2, 2.6)	1.8<br>(1.2, 2.6)	1.7<br>(1.2, 2.6)	1.7<br>(1.1, 2.6)	1.7<br>(1.1, 2.6)	1.7<br>(1.1, 2.6)	1.7<br>(1.1, 2.6)	
**70**	2.5<br>(1.6, 3.8)	2.4<br>(1.6, 3.8)	2.4<br>(1.5, 3.7)	2.4<br>(1.5, 3.7)	2.3<br>(1.5, 3.7)	2.3<br>(1.4, 3.7)	2.3<br>(1.4, 3.7)	
**75**	3.7<br>(2.4, 5.7)	3.7<br>(2.3, 5.7)	3.6<br>(2.3, 5.7)	3.6<br>(2.2, 5.7)	3.5<br>(2.2, 5.6)	3.5<br>(2.2, 5.6)	3.4<br>(2.1, 5.6)	
**80**	6.2<br>(4.0, 9.6)	6.2<br>(4.0, 9.6)	6.1<br>(3.9, 9.5)	6.0<br>(3.8, 9.5)	5.9<br>(3.7, 9.5)	5.9<br>(3.7, 9.5)	5.8<br>(3.6, 9.4)	
**85**	9.1<br>(5.6, 14.7)	9.0<br>(5.5, 14.6)	8.9<br>(5.4, 14.5)	8.8<br>(5.3, 14.4)	8.7<br>(5.2, 14.4)	8.6<br>(5.1, 14.3)	8.5<br>(5.0, 14.3)	

© 2020 Arbeev KG et al. *JAMA Network Open.*

**90**	3.0<br>(0.4, 21.3)	3.0<br>(0.4, 21.1)	3.0<br>(0.4, 20.9)	2.9<br>(0.4, 20.7)	2.9<br>(0.4, 20.5)	2.9<br>(0.4, 20.3)	2.8<br>(0.4, 20.1)	

© 2020 Arbeev KG et al. *JAMA Network Open.*

eTable 10. Hazard Ratios for All-Cause Mortality for Different Ages and Residual Leukocyte Telomere Length (rLTL) Computed From the Cox Model With Stratified Baseline Hazards for Age Groups

Age\ rLTL	-1.5	-1.4	-1.3	-1.2	-1.1	-1.0	-0.9	-0.8
**50**	1.5 (1.3, 1.8)	1.5 (1.3, 1.7)	1.4 (1.3, 1.6)	1.4 (1.3, 1.6)	1.4 (1.2, 1.5)	1.3 (1.2, 1.5)	1.3 (1.2, 1.4)	1.3 (1.2, 1.4)
**55**	2.7 (2.3, 3.2)	2.6 (2.3, 3.1)	2.6 (2.2, 3.0)	2.5 (2.2, 2.8)	2.4 (2.1, 2.7)	2.4 (2.1, 2.6)	2.3 (2.0, 2.6)	2.2 (2.0, 2.5)
**60**	4.8 (3.9, 5.9)	4.7 (3.8, 5.7)	4.5 (3.8, 5.5)	4.4 (3.7, 5.3)	4.3 (3.6, 5.1)	4.2 (3.5, 4.9)	4.0 (3.4, 4.8)	3.9 (3.3, 4.6)
**65**	8.5 (6.6, 10.9)	8.2 (6.4, 10.6)	8.0 (6.3, 10.2)	7.8 (6.1, 9.9)	7.6 (6.0, 9.6)	7.3 (5.8, 9.2)	7.1 (5.7, 8.9)	6.9 (5.5, 8.7)
**70**	15.0 (10.9, 20.5)	14.6 (10.7, 19.9)	14.2 (10.4, 19.2)	13.8 (10.2, 18.6)	13.4 (9.9, 18.0)	13.0 (9.7, 17.5)	12.6 (9.4, 16.9)	12.3 (9.2, 16.4)
**75**	26.5 (18.2, 38.6)	25.7 (17.7, 37.4)	25.0 (17.3, 36.2)	24.3 (16.8, 35.1)	23.6 (16.4, 34.0)	23.0 (16.0, 33.0)	22.3 (15.6, 32.0)	21.7 (15.2, 31.0)
**80**	55.4 (35.6, 86.3)	53.9 (34.7, 83.6)	52.4 (33.8, 81.0)	50.9 (33.0, 78.5)	49.5 (32.1, 76.1)	48.1 (31.3, 73.8)	46.7 (30.5, 71.6)	45.4 (29.7, 69.4)
**85**	98.0 (58.9, 163.0)	95.2 (57.4, 158.0)	92.5 (55.9, 153.1)	89.9 (54.5, 148.4)	87.4 (53.1, 143.9)	84.9 (51.7, 139.6)	82.5 (50.3, 135.4)	80.2 (49.0, 131.4)
**90**	173.1 (97.3, 307.9)	168.2 (94.8, 298.5)	163.5 (92.3, 289.5)	158.9 (89.9, 280.7)	154.4 (87.6, 272.3)	150.1 (85.3, 264.1)	145.8 (83.0, 256.3)	141.7 (80.8, 248.7)

Age\ rLTL	-0.7	-0.6	-0.5	-0.4	-0.3	-0.2	-0.1	0
**50**	1.2 (1.1, 1.3)	1.2 (1.1, 1.3)	1.2 (1.1, 1.2)	1.1 (1.1, 1.2)	1.1 (1.1, 1.1)	1.1 (1.0, 1.1)	1.0 (1.0, 1.0)	1.0 (1.0, 1.0)
**55**	2.2 (2.0, 2.4)	2.1 (1.9, 2.3)	2.0 (1.9, 2.2)	2.0 (1.8, 2.1)	1.9 (1.8, 2.1)	1.9 (1.7, 2.0)	1.8 (1.7, 2.0)	1.8 (1.6, 1.9)
**60**	3.8 (3.3, 4.5)	3.7 (3.2, 4.3)	3.6 (3.1, 4.2)	3.5 (3.0, 4.0)	3.4 (3.0, 3.9)	3.3 (2.9, 3.8)	3.2 (2.8, 3.7)	3.1 (2.7, 3.6)
**65**	6.7 (5.4, 8.4)	6.5 (5.3, 8.1)	6.4 (5.1, 7.9)	6.2 (5.0, 7.6)	6.0 (4.9, 7.4)	5.8 (4.7, 7.2)	5.7 (4.6, 7.0)	5.5 (4.5, 6.8)
**70**	11.9 (9.0, 15.9)	11.6 (8.7, 15.4)	11.3 (8.5, 14.9)	10.9 (8.3, 14.5)	10.6 (8.0, 14.1)	10.3 (7.8, 13.7)	10.0 (7.6, 13.3)	9.8 (7.4, 12.9)
**75**	21.1 (14.8, 30.0)	20.5 (14.4, 29.1)	19.9 (14.0, 28.3)	19.3 (13.6, 27.5)	18.8 (13.3, 26.6)	18.3 (12.9, 25.9)	17.8 (12.5, 25.1)	17.3 (12.2, 24.4)
**80**	44.1 (28.9, 67.4)	42.9 (28.1, 65.4)	41.7 (27.4, 63.4)	40.5 (26.6, 61.6)	39.4 (25.9, 59.8)	38.2 (25.2, 58.1)	37.2 (24.5, 56.4)	36.1 (23.8, 54.8)
**85**	78.0 (47.7, 127.5)	75.8 (46.4, 123.7)	73.6 (45.1, 120.1)	71.6 (43.9, 116.6)	69.5 (42.7, 113.2)	67.6 (41.5, 110.0)	65.7 (40.4, 106.8)	63.8 (39.2, 103.8)

© 2020 Arbeev KG et al. *JAMA Network Open.*

**90**	137.7 (78.6, 241.3)	133.9 (76.5, 234.3)	130.1 (74.4, 227.4)	126.4 (72.4, 220.9)	122.9 (70.4, 214.5)	119.4 (68.4, 208.3)	116.1 (66.5, 202.4)	112.8 (64.7, 196.7)

**Age\ rLTL**	**0.1**	**0.2**	**0.3**	**0.4**	**0.5**	**0.6**	**0.7**	**0.8**
**50**	1.0 (1.0, 1.0)	0.9 (0.9, 1.0)	0.9 (0.9, 0.9)	0.9 (0.9, 0.9)	0.9 (0.8, 0.9)	0.8 (0.8, 0.9)	0.8 (0.8, 0.9)	0.8 (0.7, 0.9)
**55**	1.7 (1.6, 1.8)	1.7 (1.6, 1.8)	1.6 (1.5, 1.7)	1.6 (1.5, 1.7)	1.5 (1.4, 1.7)	1.5 (1.4, 1.6)	1.4 (1.3, 1.6)	1.4 (1.3, 1.6)
**60**	3.0 (2.6, 3.5)	2.9 (2.6, 3.4)	2.9 (2.5, 3.3)	2.8 (2.4, 3.2)	2.7 (2.3, 3.1)	2.6 (2.3, 3.1)	2.6 (2.2, 3.0)	2.5 (2.1, 2.9)
**65**	5.4 (4.4, 6.6)	5.2 (4.2, 6.4)	5.1 (4.1, 6.2)	4.9 (4.0, 6.1)	4.8 (3.9, 5.9)	4.6 (3.7, 5.8)	4.5 (3.6, 5.6)	4.4 (3.5, 5.5)
**70**	9.5 (7.2, 12.5)	9.2 (7.0, 12.2)	9.0 (6.8, 11.8)	8.7 (6.6, 11.5)	8.5 (6.4, 11.2)	8.2 (6.2, 10.9)	8.0 (6.0, 10.6)	7.8 (5.8, 10.4)
**75**	16.8 (11.8, 23.7)	16.3 (11.5, 23.1)	15.8 (11.2, 22.4)	15.4 (10.9, 21.8)	15.0 (10.5, 21.2)	14.5 (10.2, 20.7)	14.1 (9.9, 20.1)	13.7 (9.6, 19.6)
**80**	35.1 (23.1, 53.3)	34.1 (22.5, 51.8)	33.2 (21.8, 50.3)	32.2 (21.2, 49.0)	31.3 (20.6, 47.6)	30.4 (20.0, 46.3)	29.6 (19.4, 45.1)	28.7 (18.8, 43.9)
**85**	62.0 (38.1, 100.9)	60.3 (37.1, 98.1)	58.6 (36.0, 95.3)	56.9 (35.0, 92.7)	55.3 (34.0, 90.2)	53.8 (33.0, 87.7)	52.3 (32.0, 85.3)	50.8 (31.1, 83.0)
**90**	109.6 (62.9, 191.1)	106.5 (61.1, 185.8)	103.5 (59.3, 180.6)	100.6 (57.6, 175.6)	97.8 (56.0, 170.7)	95.0 (54.4, 166.1)	92.3 (52.8, 161.5)	89.7 (51.2, 157.2)

**Age\ rLTL**	**0.9**	**1**	**1.1**	**1.2**	**1.3**	**1.4**	**1.5**	
**50**	0.8 (0.7, 0.8)	0.8 (0.7, 0.8)	0.7 (0.7, 0.8)	0.7 (0.6, 0.8)	0.7 (0.6, 0.8)	0.7 (0.6, 0.8)	0.7 (0.6, 0.8)	
**55**	1.4 (1.2, 1.5)	1.3 (1.2, 1.5)	1.3 (1.1, 1.5)	1.3 (1.1, 1.4)	1.2 (1.1, 1.4)	1.2 (1.0, 1.4)	1.2 (1.0, 1.3)	
**60**	2.4 (2.1, 2.8)	2.3 (2.0, 2.8)	2.3 (1.9, 2.7)	2.2 (1.9, 2.7)	2.2 (1.8, 2.6)	2.1 (1.7, 2.5)	2.0 (1.7, 2.5)	
**65**	4.3 (3.4, 5.3)	4.1 (3.3, 5.2)	4.0 (3.2, 5.1)	3.9 (3.1, 5.0)	3.8 (3.0, 4.8)	3.7 (2.9, 4.7)	3.6 (2.8, 4.6)	
**70**	7.6 (5.6, 10.1)	7.3 (5.5, 9.8)	7.1 (5.3, 9.6)	6.9 (5.1, 9.4)	6.7 (5.0, 9.1)	6.5 (4.8, 8.9)	6.4 (4.7, 8.7)	
**75**	13.3 (9.3, 19.1)	13.0 (9.1, 18.6)	12.6 (8.8, 18.1)	12.2 (8.5, 17.6)	11.9 (8.2, 17.2)	11.6 (8.0, 16.8)	11.2 (7.7, 16.3)	
**80**	27.9 (18.3, 42.7)	27.1 (17.7, 41.6)	26.4 (17.2, 40.5)	25.6 (16.7, 39.5)	24.9 (16.1, 38.4)	24.2 (15.6, 37.5)	23.5 (15.2, 36.5)	
**85**	49.4 (30.1, 80.8)	48.0 (29.2, 78.7)	46.6 (28.4, 76.6)	45.3 (27.5, 74.6)	44.0 (26.7, 72.6)	42.8 (25.9, 70.8)	41.6 (25.1, 68.9)	

© 2020 Arbeev KG et al. *JAMA Network Open.*

**90**	87.2<br>(49.7, 152.9)	84.8<br>(48.3, 148.8)	82.4<br>(46.8, 144.9)	80.1<br>(45.4, 141.1)	77.8<br>(44.1, 137.3)	75.6<br>(42.7, 133.8)	73.5<br>(41.4, 130.3)	

© 2020 Arbeev KG et al. *JAMA Network Open.*

eTable 11. Hazard Ratios for Cardiovascular Disease Mortality for Different Ages and Residual Leukocyte Telomere Length (rLTL) Computed From the Cox Model With Stratified Baseline Hazards for Age Groups

Age\ rLTL	-1.5	-1.4	-1.3	-1.2	-1.1	-1.0	-0.9	-0.8
**50**	1.4 (1.1, 1.8)	1.4 (1.1, 1.8)	1.4 (1.1, 1.7)	1.3 (1.1, 1.6)	1.3 (1.1, 1.6)	1.3 (1.1, 1.5)	1.2 (1.1, 1.4)	1.2 (1.1, 1.4)
**55**	2.8 (2.1, 3.7)	2.7 (2.1, 3.6)	2.6 (2.0, 3.4)	2.6 (2.0, 3.3)	2.5 (2.0, 3.2)	2.5 (2.0, 3.1)	2.4 (2.0, 2.9)	2.4 (1.9, 2.8)
**60**	5.4 (3.7, 7.8)	5.3 (3.7, 7.5)	5.2 (3.7, 7.3)	5.0 (3.6, 7.0)	4.9 (3.6, 6.8)	4.8 (3.5, 6.6)	4.7 (3.5, 6.3)	4.6 (3.4, 6.1)
**65**	10.5 (6.6, 16.8)	10.3 (6.5, 16.2)	10.0 (6.4, 15.7)	9.8 (6.3, 15.2)	9.6 (6.2, 14.8)	9.4 (6.1, 14.3)	9.1 (6.0, 13.9)	8.9 (5.9, 13.5)
**70**	22.2 (12.4, 39.6)	21.7 (12.2, 38.4)	21.2 (12.0, 37.2)	20.7 (11.8, 36.1)	20.2 (11.6, 35.1)	19.7 (11.4, 34.1)	19.3 (11.2, 33.1)	18.8 (11.0, 32.2)
**75**	43.2 (21.5, 86.9)	42.2 (21.1, 84.3)	41.2 (20.7, 81.9)	40.3 (20.4, 79.6)	39.3 (20.0, 77.3)	38.4 (19.6, 75.2)	37.5 (19.2, 73.1)	36.7 (18.9, 71.2)
**80**	108.2 (47.6, 245.9)	105.7 (46.8, 238.9)	103.2 (45.9, 232.2)	100.9 (45.0, 225.8)	98.5 (44.2, 219.6)	96.2 (43.3, 213.7)	94.0 (42.5, 208.0)	91.8 (41.6, 202.6)
**85**	210.8 (82.0, 542.4)	206.0 (80.4, 527.5)	201.2 (78.9, 513.0)	196.5 (77.4, 499.2)	191.9 (75.8, 485.8)	187.5 (74.3, 472.9)	183.1 (72.8, 460.6)	178.9 (71.3, 448.7)
**90**	410.8 (140.8, 1198.3)	401.3 (138.1, 1165.8)	392.0 (135.4, 1134.5)	382.9 (132.7, 1104.4)	374.0 (130.1, 1075.3)	365.3 (127.4, 1047.2)	356.8 (124.8, 1020.2)	348.6 (122.2, 994.1)

Age\ rLTL	-0.7	-0.6	-0.5	-0.4	-0.3	-0.2	-0.1	0
**50**	1.2 (1.0, 1.3)	1.2 (1.0, 1.3)	1.1 (1.0, 1.2)	1.1 (1.0, 1.2)	1.1 (1.0, 1.1)	1.0 (1.0, 1.1)	1.0 (1.0, 1.0)	1.0 (1.0, 1.0)
**55**	2.3 (1.9, 2.7)	2.2 (1.9, 2.6)	2.2 (1.9, 2.6)	2.1 (1.8, 2.5)	2.1 (1.8, 2.4)	2.0 (1.8, 2.3)	2.0 (1.7, 2.3)	1.9 (1.7, 2.2)
**60**	4.5 (3.4, 6.0)	4.4 (3.3, 5.8)	4.3 (3.2, 5.6)	4.2 (3.2, 5.5)	4.1 (3.1, 5.3)	4.0 (3.1, 5.2)	3.9 (3.0, 5.0)	3.8 (2.9, 4.9)
**65**	8.7 (5.8, 13.1)	8.5 (5.7, 12.7)	8.3 (5.6, 12.4)	8.1 (5.5, 12.1)	7.9 (5.4, 11.8)	7.8 (5.2, 11.5)	7.6 (5.1, 11.2)	7.4 (5.0, 10.9)
**70**	18.4 (10.8, 31.3)	17.9 (10.6, 30.5)	17.5 (10.4, 29.7)	17.1 (10.1, 28.9)	16.7 (9.9, 28.2)	16.3 (9.7, 27.5)	16.0 (9.5, 26.8)	15.6 (9.3, 26.2)
**75**	35.8 (18.5, 69.3)	35.0 (18.1, 67.5)	34.2 (17.7, 65.8)	33.4 (17.4, 64.1)	32.6 (17.0, 62.5)	31.8 (16.6, 61.0)	31.1 (16.2, 59.5)	30.4 (15.9, 58.1)
**80**	89.7 (40.8, 197.3)	87.6 (39.9, 192.3)	85.6 (39.1, 187.4)	83.6 (38.2, 182.7)	81.6 (37.4, 178.3)	79.7 (36.6, 173.9)	77.9 (35.7, 169.8)	76.1 (34.9, 165.8)
**85**	174.7 (69.8, 437.2)	170.7 (68.4, 426.1)	166.7 (66.9, 415.5)	162.9 (65.4, 405.3)	159.1 (64.0, 395.4)	155.4 (62.6, 385.9)	151.8 (61.1, 376.7)	148.3 (59.7, 367.9)

© 2020 Arbeev KG et al. *JAMA Network Open.*

90	340.5 (119.6, 969.0)	332.6 (117.1, 944.7)	324.8 (114.5, 921.4)	317.3 (112.0, 898.8)	309.9 (109.5, 877.1)	302.7 (107.1, 856.1)	295.7 (104.6, 835.8)	288.9 (102.2, 816.3)

Age\ rLTL	0.1	0.2	0.3	0.4	0.5	0.6	0.7	0.8
50	1.0 (1.0, 1.0)	1.0 (0.9, 1.0)	0.9 (0.9, 1.0)	0.9 (0.9, 1.0)	0.9 (0.8, 1.0)	0.9 (0.8, 1.0)	0.8 (0.8, 1.0)	0.8 (0.7, 0.9)
55	1.9 (1.7, 2.2)	1.9 (1.6, 2.1)	1.8 (1.6, 2.1)	1.8 (1.5, 2.1)	1.7 (1.5, 2.0)	1.7 (1.4, 2.0)	1.7 (1.4, 2.0)	1.6 (1.3, 1.9)
60	3.7 (2.9, 4.8)	3.6 (2.8, 4.7)	3.5 (2.7, 4.6)	3.5 (2.6, 4.5)	3.4 (2.6, 4.4)	3.3 (2.5, 4.4)	3.2 (2.4, 4.3)	3.1 (2.3, 4.2)
65	7.2 (4.9, 10.7)	7.1 (4.8, 10.4)	6.9 (4.7, 10.2)	6.7 (4.5, 10.0)	6.6 (4.4, 9.8)	6.4 (4.3, 9.6)	6.3 (4.2, 9.4)	6.1 (4.1, 9.3)
70	15.2 (9.1, 25.6)	14.9 (8.8, 25.0)	14.5 (8.6, 24.5)	14.2 (8.4, 24.0)	13.9 (8.2, 23.5)	13.5 (8.0, 23.0)	13.2 (7.8, 22.5)	12.9 (7.6, 22.1)
75	29.7 (15.5, 56.8)	29.0 (15.1, 55.5)	28.3 (14.8, 54.3)	27.7 (14.4, 53.1)	27.0 (14.0, 52.0)	26.4 (13.7, 50.9)	25.8 (13.3, 49.9)	25.2 (13.0, 48.9)
80	74.3 (34.1, 162.0)	72.6 (33.3, 158.3)	70.9 (32.5, 154.8)	69.3 (31.7, 151.4)	67.7 (30.9, 148.1)	66.1 (30.1, 145.0)	64.6 (29.4, 141.9)	63.1 (28.6, 139.0)
85	144.8 (58.3, 359.4)	141.4 (57.0, 351.2)	138.2 (55.6, 343.3)	135.0 (54.3, 335.7)	131.8 (52.9, 328.4)	128.8 (51.6, 321.3)	125.8 (50.3, 314.5)	122.9 (49.0, 307.9)
90	282.2 (99.8, 797.4)	275.6 (97.5, 779.2)	269.2 (95.2, 761.6)	263.0 (92.9, 744.6)	256.9 (90.6, 728.2)	250.9 (88.4, 712.4)	245.1 (86.2, 697.1)	239.4 (84.0, 682.3)

Age\ rLTL	0.9	1	1.1	1.2	1.3	1.4	1.5	
50	0.8 (0.7, 0.9)	0.8 (0.7, 0.9)	0.8 (0.6, 0.9)	0.8 (0.6, 0.9)	0.7 (0.6, 0.9)	0.7 (0.6, 0.9)	0.7 (0.5, 0.9)	
55	1.6 (1.3, 1.9)	1.5 (1.2, 1.9)	1.5 (1.2, 1.9)	1.5 (1.2, 1.9)	1.4 (1.1, 1.9)	1.4 (1.1, 1.8)	1.4 (1.0, 1.8)	
60	3.1 (2.3, 4.2)	3.0 (2.2, 4.1)	2.9 (2.1, 4.0)	2.9 (2.1, 4.0)	2.8 (2.0, 3.9)	2.7 (1.9, 3.9)	2.7 (1.9, 3.8)	
65	6.0 (3.9, 9.1)	5.8 (3.8, 8.9)	5.7 (3.7, 8.8)	5.6 (3.6, 8.7)	5.5 (3.5, 8.5)	5.3 (3.4, 8.4)	5.2 (3.3, 8.3)	
70	12.6 (7.3, 21.7)	12.3 (7.1, 21.3)	12.0 (6.9, 20.9)	11.8 (6.7, 20.5)	11.5 (6.5, 20.2)	11.2 (6.3, 19.9)	11.0 (6.1, 19.5)	
75	24.6 (12.6, 47.9)	24.0 (12.3, 47.0)	23.5 (11.9, 46.1)	22.9 (11.6, 45.2)	22.4 (11.3, 44.4)	21.9 (11.0, 43.6)	21.4 (10.6, 42.9)	
80	61.6 (27.9, 136.2)	60.2 (27.1, 133.5)	58.8 (26.4, 130.9)	57.4 (25.7, 128.4)	56.1 (25.0, 126.0)	54.8 (24.3, 123.6)	53.5 (23.6, 121.4)	
85	120.0 (47.8, 301.6)	117.2 (46.5, 295.4)	114.5 (45.3, 289.5)	111.8 (44.1, 283.8)	109.3 (42.9, 278.3)	106.7 (41.7, 273.0)	104.2 (40.6, 267.8)	

© 2020 Arbeev KG et al. *JAMA Network Open.*

**90**	233.8<br>(81.9, 668.0)	228.4<br>(79.7, 654.2)	223.1<br>(77.7, 640.8)	217.9<br>(75.6, 627.9)	212.9<br>(73.6, 615.4)	207.9<br>(71.7, 603.4)	203.1<br>(69.7, 591.7)	

© 2020 Arbeev KG et al. *JAMA Network Open.*

eTable 12. Hazard Ratios for Other Causes Mortality for Different Ages and Residual Leukocyte Telomere Length (rLTL) Computed From the Cox Model With Stratified Baseline Hazards for Age Groups

Age\ rLTL	-1.5	-1.4	-1.3	-1.2	-1.1	-1.0	-0.9	-0.8
50	1.9 (1.5, 2.3)	1.8 (1.5, 2.2)	1.7 (1.4, 2.1)	1.7 (1.4, 2.0)	1.6 (1.4, 1.9)	1.5 (1.3, 1.8)	1.5 (1.3, 1.7)	1.4 (1.2, 1.6)
55	4.1 (3.2, 5.3)	3.9 (3.1, 5.0)	3.8 (3.0, 4.7)	3.6 (2.9, 4.5)	3.5 (2.8, 4.2)	3.3 (2.8, 4.0)	3.2 (2.7, 3.8)	3.1 (2.6, 3.6)
60	8.9 (6.5, 12.2)	8.6 (6.3, 11.6)	8.2 (6.1, 11.0)	7.9 (5.9, 10.5)	7.5 (5.7, 10.0)	7.2 (5.5, 9.5)	6.9 (5.3, 9.0)	6.6 (5.1, 8.6)
65	19.4 (13.0, 29.1)	18.6 (12.5, 27.7)	17.8 (12.1, 26.3)	17.1 (11.7, 25.1)	16.4 (11.3, 23.9)	15.7 (10.9, 22.8)	15.1 (10.5, 21.7)	14.5 (10.1, 20.7)
70	35.3 (21.4, 58.4)	33.9 (20.6, 55.7)	32.5 (19.9, 53.0)	31.1 (19.2, 50.6)	29.9 (18.5, 48.2)	28.6 (17.8, 46.0)	27.4 (17.2, 43.9)	26.3 (16.5, 41.9)
75	76.9 (42.0, 141.0)	73.8 (40.5, 134.4)	70.7 (39.0, 128.2)	67.8 (37.6, 122.3)	65.0 (36.2, 116.8)	62.3 (34.8, 111.5)	59.7 (33.5, 106.5)	57.3 (32.2, 101.8)
80	124.6 (61.2, 253.8)	119.5 (58.9, 242.2)	114.5 (56.7, 231.2)	109.8 (54.6, 220.7)	105.3 (52.6, 210.8)	100.9 (50.6, 201.4)	96.8 (48.6, 192.5)	92.8 (46.8, 184.0)
85	271.3 (119.6, 615.1)	260.1 (115.2, 587.3)	249.3 (110.8, 561.0)	239.0 (106.6, 535.9)	229.2 (102.6, 512.1)	219.7 (98.6, 489.5)	210.6 (94.8, 468.0)	201.9 (91.1, 447.5)
90	590.5 (233.6, 1492.6)	566.1 (224.8, 1425.8)	542.8 (216.2, 1362.4)	520.4 (208.0, 1302.0)	498.9 (199.9, 1244.7)	478.3 (192.2, 1190.1)	458.5 (184.7, 1138.2)	439.6 (177.5, 1088.8)

Age\ rLTL	-0.7	-0.6	-0.5	-0.4	-0.3	-0.2	-0.1	0
50	1.3 (1.2, 1.5)	1.3 (1.2, 1.4)	1.2 (1.1, 1.3)	1.2 (1.1, 1.3)	1.1 (1.1, 1.2)	1.1 (1.1, 1.1)	1.0 (1.0, 1.1)	1.0 (1.0, 1.0)
55	2.9 (2.5, 3.4)	2.8 (2.4, 3.2)	2.7 (2.3, 3.1)	2.6 (2.3, 2.9)	2.5 (2.2, 2.8)	2.4 (2.1, 2.7)	2.3 (2.0, 2.5)	2.2 (1.9, 2.4)
60	6.4 (5.0, 8.2)	6.1 (4.8, 7.8)	5.9 (4.6, 7.4)	5.6 (4.4, 7.1)	5.4 (4.3, 6.8)	5.2 (4.1, 6.5)	4.9 (3.9, 6.2)	4.7 (3.8, 5.9)
65	13.9 (9.7, 19.7)	13.3 (9.4, 18.8)	12.7 (9.0, 18.0)	12.2 (8.7, 17.2)	11.7 (8.3, 16.5)	11.2 (8.0, 15.7)	10.8 (7.7, 15.1)	10.3 (7.4, 14.4)
70	25.2 (15.9, 40.0)	24.2 (15.3, 38.2)	23.2 (14.7, 36.6)	22.2 (14.1, 35.0)	21.3 (13.6, 33.5)	20.4 (13.0, 32.0)	19.6 (12.5, 30.7)	18.8 (12.0, 29.4)
75	54.9 (31.0, 97.3)	52.6 (29.8, 93.0)	50.5 (28.6, 89.0)	48.4 (27.5, 85.1)	46.4 (26.4, 81.5)	44.5 (25.3, 78.0)	42.6 (24.3, 74.7)	40.9 (23.3, 71.6)
80	88.9 (44.9, 176.0)	85.3 (43.2, 168.3)	81.7 (41.5, 161.1)	78.4 (39.8, 154.2)	75.1 (38.2, 147.6)	72.0 (36.7, 141.4)	69.0 (35.2, 135.5)	66.2 (33.8, 129.8)
85	193.6 (87.5, 428.1)	185.6 (84.1, 409.6)	177.9 (80.8, 392.1)	170.6 (77.5, 375.3)	163.5 (74.4, 359.4)	156.8 (71.4, 344.3)	150.3 (68.5, 329.9)	144.1 (65.7, 316.2)

© 2020 Arbeev KG et al. *JAMA Network Open.*

**90**	421.4<br>(170.5, 1041.8)	404.0<br>(163.7, 997.0)	387.3<br>(157.2, 954.4)	371.3<br>(150.9, 913.9)	356.0<br>(144.8, 875.3)	341.3<br>(138.9, 838.5)	327.2<br>(133.3, 803.4)	313.7<br>(127.8, 770.0)

Age\ rLTL	0.1	0.2	0.3	0.4	0.5	0.6	0.7	0.8
**50**	1.0<br>(0.9, 1.0)	0.9<br>(0.9, 0.9)	0.9<br>(0.8, 0.9)	0.8<br>(0.8, 0.9)	0.8<br>(0.8, 0.9)	0.8<br>(0.7, 0.8)	0.7<br>(0.7, 0.8)	0.7<br>(0.6, 0.8)
**55**	2.1<br>(1.9, 2.3)	2.0<br>(1.8, 2.2)	1.9<br>(1.7, 2.2)	1.8<br>(1.6, 2.1)	1.8<br>(1.5, 2.0)	1.7<br>(1.5, 1.9)	1.6<br>(1.4, 1.9)	1.6<br>(1.3, 1.8)
**60**	4.5<br>(3.6, 5.7)	4.4<br>(3.5, 5.5)	4.2<br>(3.3, 5.2)	4.0<br>(3.2, 5.0)	3.8<br>(3.0, 4.9)	3.7<br>(2.9, 4.7)	3.5<br>(2.8, 4.5)	3.4<br>(2.6, 4.4)
**65**	9.9<br>(7.1, 13.9)	9.5<br>(6.8, 13.3)	9.1<br>(6.5, 12.8)	8.7<br>(6.2, 12.3)	8.4<br>(5.9, 11.8)	8.0<br>(5.7, 11.3)	7.7<br>(5.4, 10.9)	7.4<br>(5.2, 10.5)
**70**	18.0<br>(11.5, 28.2)	17.3<br>(11.0, 27.1)	16.5<br>(10.5, 26.0)	15.9<br>(10.1, 24.9)	15.2<br>(9.7, 23.9)	14.6<br>(9.2, 23.0)	14.0<br>(8.8, 22.1)	13.4<br>(8.4, 21.3)
**75**	39.2<br>(22.4, 68.7)	37.6<br>(21.4, 65.9)	36.0<br>(20.5, 63.2)	34.5<br>(19.6, 60.7)	33.1<br>(18.8, 58.3)	31.7<br>(18.0, 56.0)	30.4<br>(17.2, 53.8)	29.2<br>(16.5, 51.7)
**80**	63.5<br>(32.4, 124.5)	60.8<br>(31.0, 119.4)	58.3<br>(29.7, 114.5)	55.9<br>(28.5, 109.9)	53.6<br>(27.2, 105.5)	51.4<br>(26.1, 101.3)	49.3<br>(25.0, 97.3)	47.2<br>(23.9, 93.5)
**85**	138.2<br>(63.0, 303.1)	132.4<br>(60.4, 290.7)	127.0<br>(57.8, 278.8)	121.7<br>(55.4, 267.5)	116.7<br>(53.1, 256.8)	111.9<br>(50.8, 246.5)	107.3<br>(48.6, 236.7)	102.8<br>(46.5, 227.4)
**90**	300.7<br>(122.5, 738.2)	288.3<br>(117.4, 707.9)	276.4<br>(112.5, 679.0)	265.0<br>(107.8, 651.4)	254.1<br>(103.3, 625.1)	243.6<br>(98.9, 600.0)	233.5<br>(94.7, 576.0)	223.9<br>(90.6, 553.1)

Age\ rLTL	0.9	1	1.1	1.2	1.3	1.4	1.5	
**50**	0.7<br>(0.6, 0.8)	0.7<br>(0.6, 0.8)	0.6<br>(0.5, 0.7)	0.6<br>(0.5, 0.7)	0.6<br>(0.5, 0.7)	0.6<br>(0.5, 0.7)	0.5<br>(0.4, 0.7)	
**55**	1.5<br>(1.3, 1.8)	1.4<br>(1.2, 1.7)	1.4<br>(1.1, 1.7)	1.3<br>(1.1, 1.6)	1.3<br>(1.0, 1.6)	1.2<br>(1.0, 1.5)	1.2<br>(0.9, 1.5)	
**60**	3.2<br>(2.5, 4.2)	3.1<br>(2.4, 4.1)	3.0<br>(2.3, 3.9)	2.9<br>(2.1, 3.8)	2.7<br>(2.0, 3.7)	2.6<br>(1.9, 3.6)	2.5<br>(1.8, 3.4)	
**65**	7.1<br>(4.9, 10.1)	6.8<br>(4.7, 9.8)	6.5<br>(4.5, 9.4)	6.2<br>(4.3, 9.1)	6.0<br>(4.1, 8.8)	5.7<br>(3.9, 8.5)	5.5<br>(3.7, 8.2)	
**70**	12.8<br>(8.1, 20.5)	12.3<br>(7.7, 19.7)	11.8<br>(7.3, 19.0)	11.3<br>(7.0, 18.3)	10.9<br>(6.7, 17.6)	10.4<br>(6.4, 17.0)	10.0<br>(6.1, 16.4)	
**75**	28.0<br>(15.7, 49.7)	26.8<br>(15.0, 47.8)	25.7<br>(14.4, 46.0)	24.6<br>(13.7, 44.3)	23.6<br>(13.1, 42.7)	22.6<br>(12.5, 41.1)	21.7<br>(11.9, 39.6)	
**80**	45.3<br>(22.8, 89.9)	43.4<br>(21.8, 86.4)	41.6<br>(20.9, 83.1)	39.9<br>(19.9, 79.9)	38.3<br>(19.0, 76.9)	36.7<br>(18.2, 74.0)	35.2<br>(17.3, 71.3)	
**85**	98.6<br>(44.5, 218.4)	94.5<br>(42.6, 209.9)	90.6<br>(40.7, 201.8)	86.9<br>(38.9, 194.1)	83.3<br>(37.2, 186.6)	79.8<br>(35.5, 179.6)	76.6<br>(33.9, 172.8)	

© 2020 Arbeev KG et al. *JAMA Network Open.*

**90**	214.6<br>(86.7, 531.3)	205.8<br>(82.9, 510.4)	197.3<br>(79.3, 490.5)	189.1<br>(75.9, 471.5)	181.3<br>(72.5, 453.3)	173.8<br>(69.3, 435.9)	166.6<br>(66.2, 419.3)	

© 2020 Arbeev KG et al. *JAMA Network Open.*

eTable 13. Hazard Ratios for Cancer Mortality for Different Ages and Residual Leukocyte Telomere Length (rLTL) Computed From the Cox Model With Stratified Baseline Hazards for Age Groups

Age\ rLTL	-1.5	-1.4	-1.3	-1.2	-1.1	-1.0	-0.9	-0.8
**50**	1.2 (0.9, 1.6)	1.2 (0.9, 1.5)	1.2 (0.9, 1.5)	1.1 (0.9, 1.4)	1.1 (0.9, 1.4)	1.1 (0.9, 1.4)	1.1 (0.9, 1.3)	1.1 (0.9, 1.3)
**55**	1.5 (1.1, 2.0)	1.5 (1.1, 2.0)	1.4 (1.1, 1.9)	1.4 (1.1, 1.9)	1.4 (1.1, 1.8)	1.4 (1.1, 1.8)	1.4 (1.1, 1.7)	1.4 (1.1, 1.7)
**60**	1.8 (1.3, 2.7)	1.8 (1.3, 2.6)	1.8 (1.3, 2.6)	1.8 (1.3, 2.5)	1.8 (1.3, 2.4)	1.7 (1.3, 2.4)	1.7 (1.3, 2.3)	1.7 (1.3, 2.3)
**65**	2.3 (1.5, 3.7)	2.3 (1.5, 3.6)	2.3 (1.5, 3.5)	2.2 (1.5, 3.4)	2.2 (1.5, 3.3)	2.2 (1.5, 3.3)	2.2 (1.5, 3.2)	2.1 (1.4, 3.2)
**70**	4.9 (2.8, 8.6)	4.9 (2.8, 8.4)	4.8 (2.8, 8.2)	4.8 (2.8, 8.1)	4.7 (2.8, 7.9)	4.7 (2.8, 7.8)	4.6 (2.8, 7.6)	4.6 (2.8, 7.5)
**75**	6.2 (3.2, 11.9)	6.1 (3.2, 11.7)	6.0 (3.2, 11.5)	5.9 (3.1, 11.3)	5.9 (3.1, 11.1)	5.8 (3.1, 10.9)	5.8 (3.1, 10.7)	5.7 (3.1, 10.5)
**80**	6.5 (3.0, 14.1)	6.5 (3.0, 13.9)	6.4 (3.0, 13.6)	6.3 (3.0, 13.4)	6.3 (3.0, 13.2)	6.2 (3.0, 13.0)	6.1 (2.9, 12.7)	6.0 (2.9, 12.6)
**85**	8.2 (3.4, 19.7)	8.1 (3.4, 19.4)	8.0 (3.4, 19.1)	7.9 (3.3, 18.8)	7.8 (3.3, 18.5)	7.7 (3.3, 18.2)	7.6 (3.3, 17.9)	7.6 (3.2, 17.6)
**90**	10.2 (3.8, 27.6)	10.1 (3.8, 27.2)	10.0 (3.7, 26.7)	9.9 (3.7, 26.3)	9.8 (3.7, 25.9)	9.7 (3.7, 25.5)	9.6 (3.6, 25.1)	9.4 (3.6, 24.7)

Age\ rLTL	-0.7	-0.6	-0.5	-0.4	-0.3	-0.2	-0.1	0
**50**	1.1 (0.9, 1.2)	1.1 (1.0, 1.2)	1.1 (1.0, 1.2)	1.0 (1.0, 1.1)	1.0 (1.0, 1.1)	1.0 (1.0, 1.1)	1.0 (1.0, 1.0)	1.0 (1.0, 1.0)
**55**	1.4 (1.1, 1.6)	1.3 (1.1, 1.6)	1.3 (1.1, 1.5)	1.3 (1.1, 1.5)	1.3 (1.1, 1.5)	1.3 (1.1, 1.4)	1.3 (1.1, 1.4)	1.2 (1.1, 1.4)
**60**	1.7 (1.3, 2.2)	1.7 (1.3, 2.2)	1.7 (1.3, 2.1)	1.6 (1.3, 2.1)	1.6 (1.3, 2.1)	1.6 (1.3, 2.0)	1.6 (1.2, 2.0)	1.6 (1.2, 2.0)
**65**	2.1 (1.4, 3.1)	2.1 (1.4, 3.0)	2.1 (1.4, 3.0)	2.0 (1.4, 2.9)	2.0 (1.4, 2.9)	2.0 (1.4, 2.9)	2.0 (1.4, 2.8)	2.0 (1.4, 2.8)
**70**	4.5 (2.7, 7.4)	4.4 (2.7, 7.3)	4.4 (2.7, 7.1)	4.4 (2.7, 7.0)	4.3 (2.7, 6.9)	4.3 (2.6, 6.8)	4.2 (2.6, 6.8)	4.2 (2.6, 6.7)
**75**	5.6 (3.1, 10.3)	5.6 (3.0, 10.2)	5.5 (3.0, 10.0)	5.4 (3.0, 9.9)	5.4 (3.0, 9.8)	5.3 (2.9, 9.6)	5.3 (2.9, 9.5)	5.2 (2.9, 9.4)
**80**	6.0 (2.9, 12.4)	5.9 (2.9, 12.2)	5.8 (2.8, 12.0)	5.8 (2.8, 11.8)	5.7 (2.8, 11.7)	5.7 (2.8, 11.5)	5.6 (2.7, 11.4)	5.5 (2.7, 11.3)
**85**	7.5 (3.2, 17.4)	7.4 (3.2, 17.1)	7.3 (3.2, 16.9)	7.2 (3.1, 16.7)	7.1 (3.1, 16.4)	7.1 (3.1, 16.2)	7.0 (3.0, 16.0)	6.9 (3.0, 15.8)

© 2020 Arbeev KG et al. *JAMA Network Open.*

**90**	9.3<br>(3.6, 24.4)	9.2<br>(3.5, 24.1)	9.1<br>(3.5, 23.7)	9.0<br>(3.5, 23.4)	8.9<br>(3.4, 23.1)	8.8<br>(3.4, 22.8)	8.7<br>(3.4, 22.6)	8.6<br>(3.3, 22.3)

Age\ rLTL	0.1	0.2	0.3	0.4	0.5	0.6	0.7	0.8
**50**	1.0<br>(1.0, 1.0)	1.0<br>(0.9, 1.0)	1.0<br>(0.9, 1.0)	1.0<br>(0.9, 1.0)	0.9<br>(0.9, 1.0)	0.9<br>(0.8, 1.0)	0.9<br>(0.8, 1.1)	0.9<br>(0.8, 1.1)
**55**	1.2<br>(1.1, 1.4)	1.2<br>(1.1, 1.4)	1.2<br>(1.1, 1.4)	1.2<br>(1.0, 1.4)	1.2<br>(1.0, 1.4)	1.2<br>(1.0, 1.4)	1.2<br>(1.0, 1.4)	1.1<br>(0.9, 1.4)
**60**	1.5<br>(1.2, 2.0)	1.5<br>(1.2, 1.9)	1.5<br>(1.2, 1.9)	1.5<br>(1.2, 1.9)	1.5<br>(1.1, 1.9)	1.5<br>(1.1, 1.9)	1.4<br>(1.1, 1.9)	1.4<br>(1.1, 1.9)
**65**	1.9<br>(1.4, 2.8)	1.9<br>(1.3, 2.7)	1.9<br>(1.3, 2.7)	1.9<br>(1.3, 2.7)	1.8<br>(1.3, 2.7)	1.8<br>(1.3, 2.6)	1.8<br>(1.2, 2.6)	1.8<br>(1.2, 2.6)
**70**	4.1<br>(2.6, 6.6)	4.1<br>(2.5, 6.5)	4.0<br>(2.5, 6.5)	4.0<br>(2.5, 6.4)	3.9<br>(2.4, 6.4)	3.9<br>(2.4, 6.3)	3.8<br>(2.4, 6.3)	3.8<br>(2.3, 6.2)
**75**	5.1<br>(2.8, 9.3)	5.1<br>(2.8, 9.2)	5.0<br>(2.8, 9.1)	5.0<br>(2.7, 9.0)	4.9<br>(2.7, 8.9)	4.9<br>(2.7, 8.9)	4.8<br>(2.6, 8.8)	4.7<br>(2.6, 8.7)
**80**	5.5<br>(2.7, 11.1)	5.4<br>(2.7, 11.0)	5.3<br>(2.6, 10.9)	5.3<br>(2.6, 10.8)	5.2<br>(2.6, 10.7)	5.2<br>(2.5, 10.6)	5.1<br>(2.5, 10.5)	5.1<br>(2.4, 10.4)
**85**	6.8<br>(3.0, 15.7)	6.8<br>(2.9, 15.5)	6.7<br>(2.9, 15.3)	6.6<br>(2.9, 15.2)	6.5<br>(2.8, 15.0)	6.5<br>(2.8, 14.9)	6.4<br>(2.8, 14.8)	6.3<br>(2.7, 14.6)
**90**	8.5<br>(3.3, 22.0)	8.4<br>(3.3, 21.8)	8.3<br>(3.2, 21.6)	8.3<br>(3.2, 21.3)	8.2<br>(3.2, 21.1)	8.1<br>(3.1, 20.9)	8.0<br>(3.1, 20.7)	7.9<br>(3.0, 20.6)

Age\ rLTL	0.9	1	1.1	1.2	1.3	1.4	1.5	
**50**	0.9<br>(0.8, 1.1)	0.9<br>(0.7, 1.1)	0.9<br>(0.7, 1.1)	0.9<br>(0.7, 1.1)	0.9<br>(0.7, 1.1)	0.9<br>(0.7, 1.1)	0.8<br>(0.6, 1.1)	
**55**	1.1<br>(0.9, 1.4)	1.1<br>(0.9, 1.4)	1.1<br>(0.9, 1.4)	1.1<br>(0.8, 1.4)	1.1<br>(0.8, 1.4)	1.1<br>(0.8, 1.4)	1.1<br>(0.8, 1.4)	
**60**	1.4<br>(1.1, 1.9)	1.4<br>(1.0, 1.9)	1.4<br>(1.0, 1.9)	1.4<br>(1.0, 1.9)	1.3<br>(1.0, 1.9)	1.3<br>(0.9, 1.9)	1.3<br>(0.9, 1.9)	
**65**	1.8<br>(1.2, 2.6)	1.7<br>(1.2, 2.6)	1.7<br>(1.1, 2.6)	1.7<br>(1.1, 2.6)	1.7<br>(1.1, 2.6)	1.7<br>(1.1, 2.6)	1.6<br>(1.1, 2.6)	
**70**	3.8<br>(2.3, 6.2)	3.7<br>(2.2, 6.2)	3.7<br>(2.2, 6.1)	3.6<br>(2.2, 6.1)	3.6<br>(2.1, 6.1)	3.6<br>(2.1, 6.1)	3.5<br>(2.0, 6.1)	
**75**	4.7<br>(2.5, 8.7)	4.6<br>(2.5, 8.6)	4.6<br>(2.5, 8.6)	4.5<br>(2.4, 8.5)	4.5<br>(2.4, 8.5)	4.4<br>(2.3, 8.5)	4.4<br>(2.3, 8.4)	
**80**	5.0<br>(2.4, 10.4)	4.9<br>(2.4, 10.3)	4.9<br>(2.3, 10.2)	4.8<br>(2.3, 10.2)	4.8<br>(2.3, 10.1)	4.7<br>(2.2, 10.0)	4.7<br>(2.2, 10.0)	
**85**	6.2<br>(2.7, 14.5)	6.2<br>(2.6, 14.4)	6.1<br>(2.6, 14.3)	6.0<br>(2.6, 14.2)	6.0<br>(2.5, 14.1)	5.9<br>(2.5, 14.0)	5.8<br>(2.4, 14.0)	

© 2020 Arbeev KG et al. *JAMA Network Open.*

**90**	7.8<br>(3.0, 20.4)	7.7<br>(2.9, 20.2)	7.6<br>(2.9, 20.1)	7.5<br>(2.9, 19.9)	7.5<br>(2.8, 19.8)	7.4<br>(2.8, 19.7)	7.3<br>(2.7, 19.5)	

© 2020 Arbeev KG et al. *JAMA Network Open.*

eFigure 1. Hazard Ratios for All-Cause and Cause-Specific Mortality for Different Residual Leukocyte Telomere Lengths

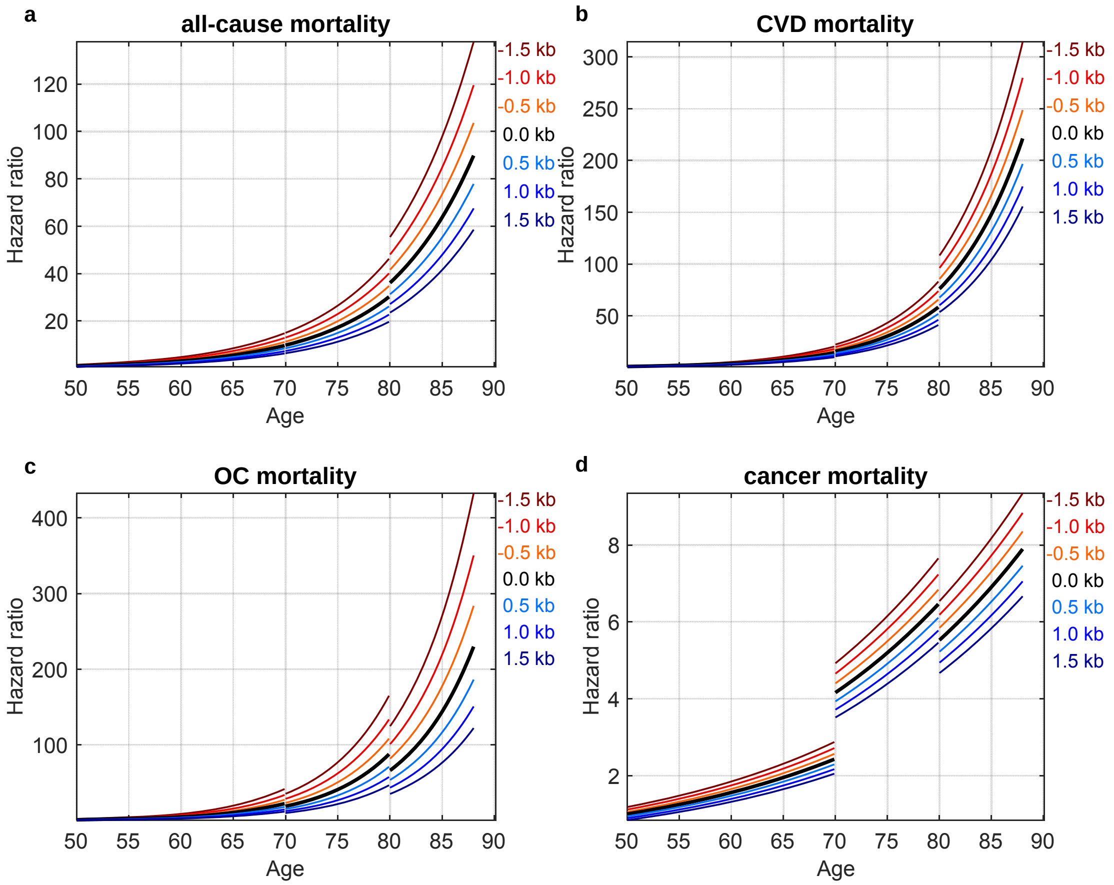

The figure is similar to Figure 2 in main text but it is for the model with stratified baseline hazards for age groups.

© 2020 Arbeev KG et al. *JAMA Network Open.*

eFigure 2. Hazard Ratios for All-Cause and Cause-Specific Mortality at Different Ages

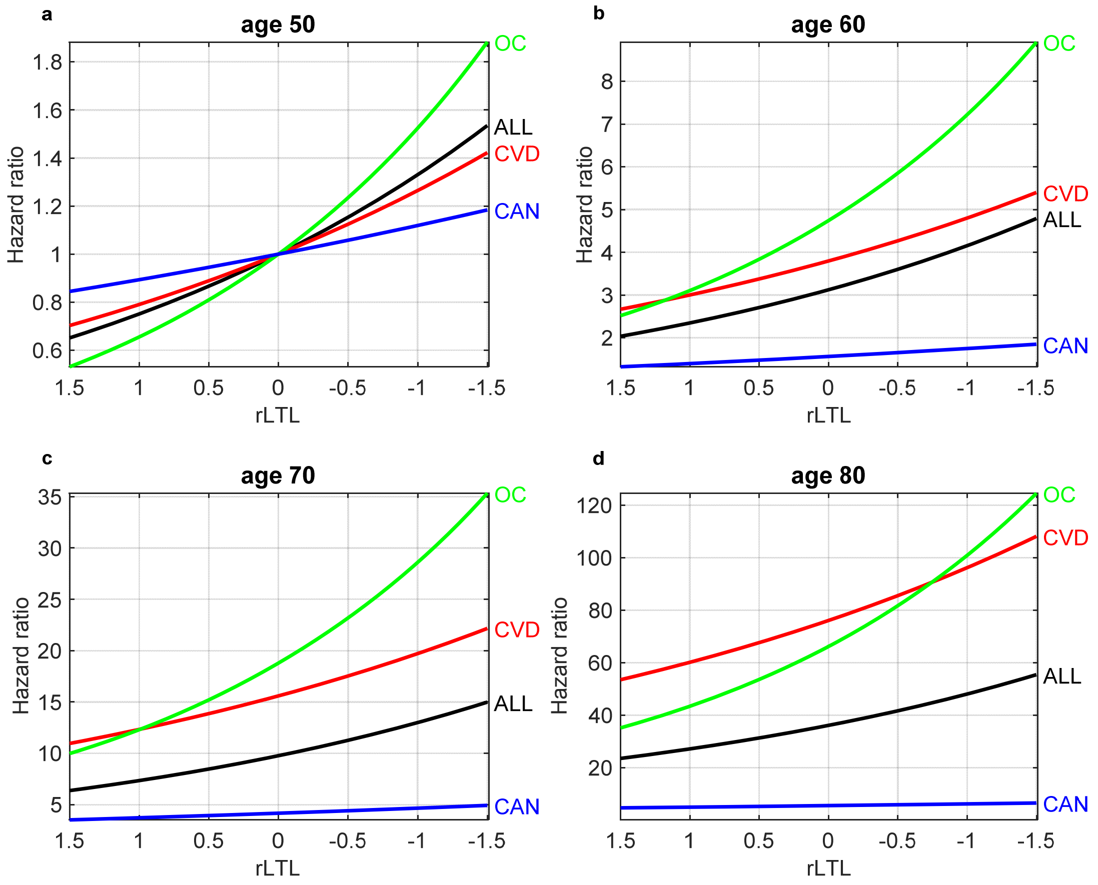

The figure is similar to Figure 3 in main text but it is for the model with stratified baseline hazards for age groups.

© 2020 Arbeev KG et al. *JAMA Network Open.*

eFigure 3. Hazard Ratios for Noncancer Mortality for Different Ages and Values of Residual Leukocyte Telomere Length

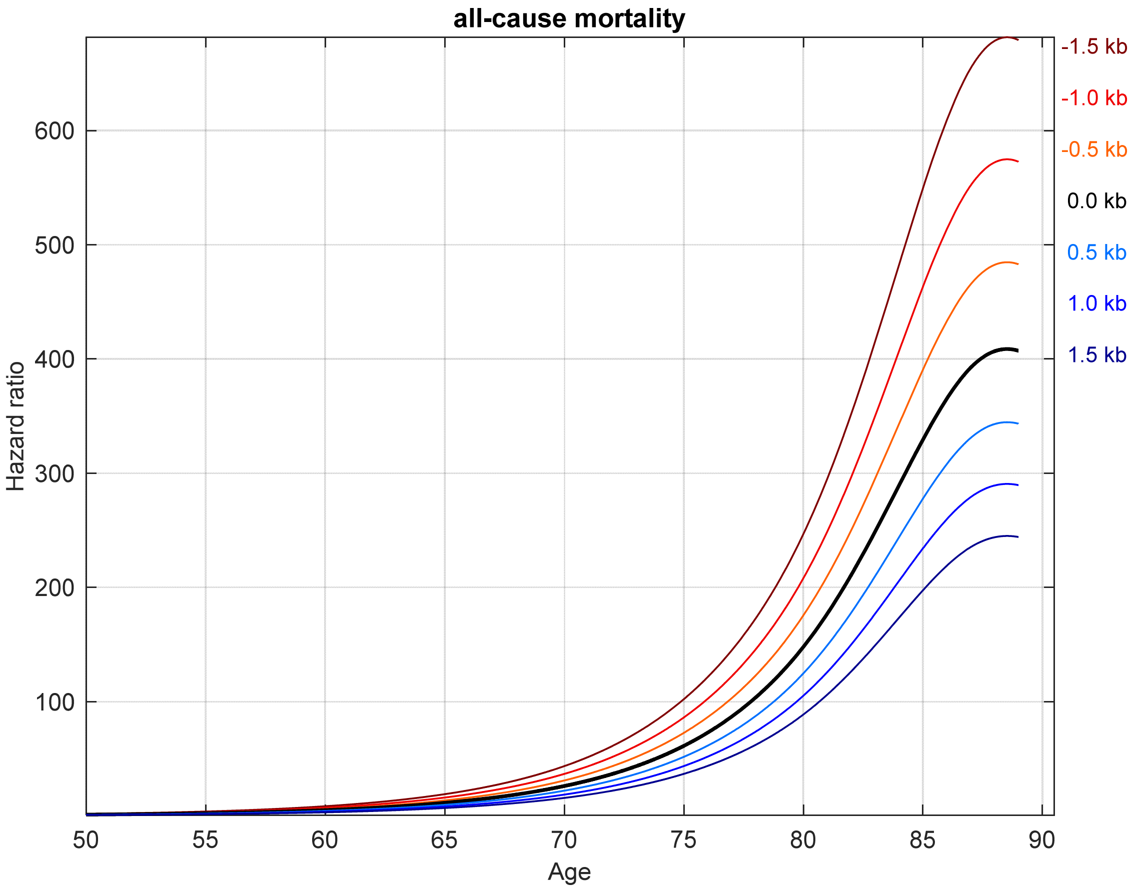

The figure is similar to Figure 2 (a) in main text but with cancer deaths removed.

© 2020 Arbeev KG et al. *JAMA Network Open.*

eFigure 4. Hazard Ratios at Different Ages for Noncancer Mortality in the Model with Splines for Age

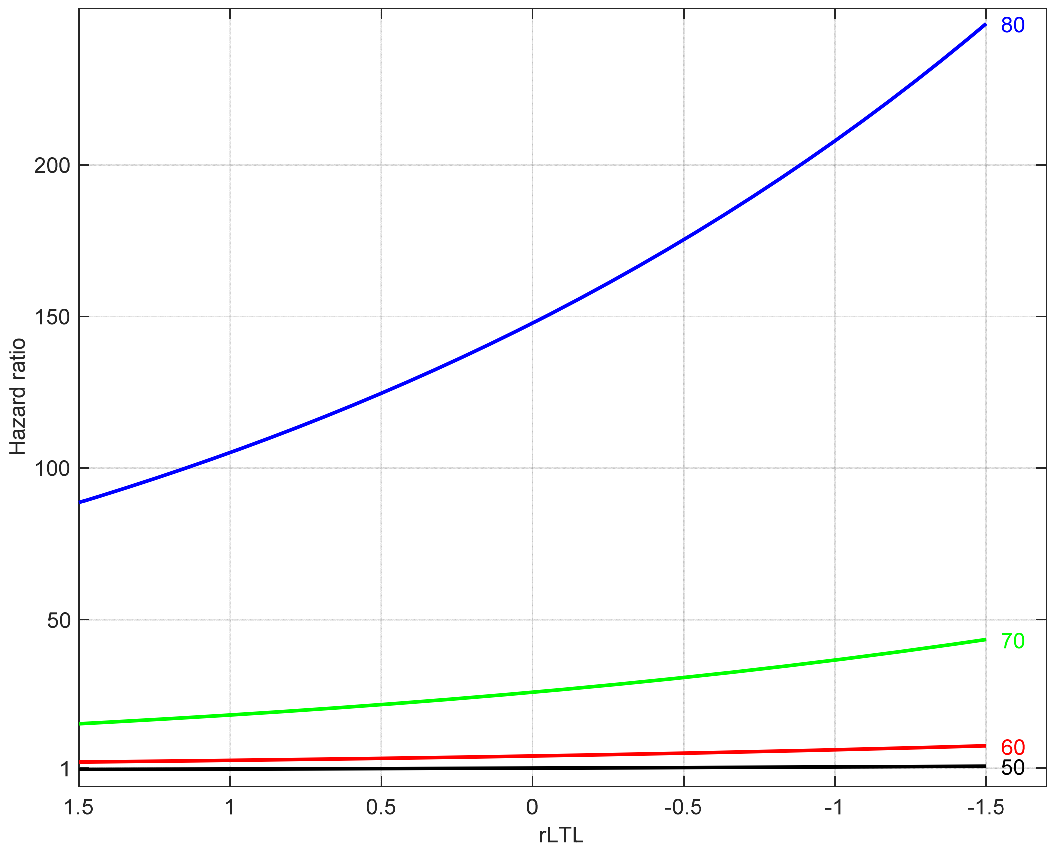

The figure shows the lines for all-cause mortality (as in Figure 3) but with cancer deaths removed.

© 2020 Arbeev KG et al. *JAMA Network Open.*

eFigure 5. Hazard Ratios for Noncancer Mortality for Different Ages and Values of Residual Leukocyte Telomere Length

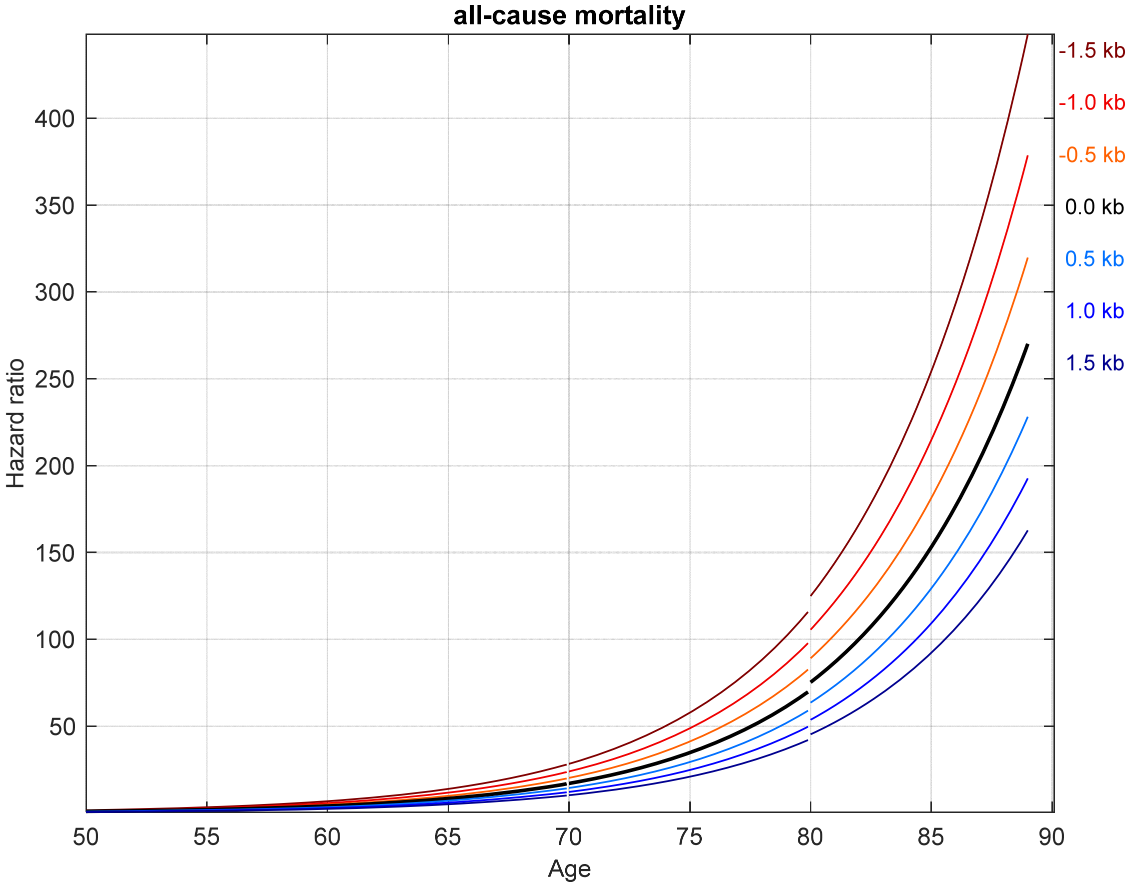

The figure is similar to eFigure 3 but it is for the model with stratified baseline hazards for age groups.

© 2020 Arbeev KG et al. *JAMA Network Open.*

eFigure 6. Hazard Ratios at Different Ages for Noncancer Mortality in the Model With Stratified Baseline Hazards

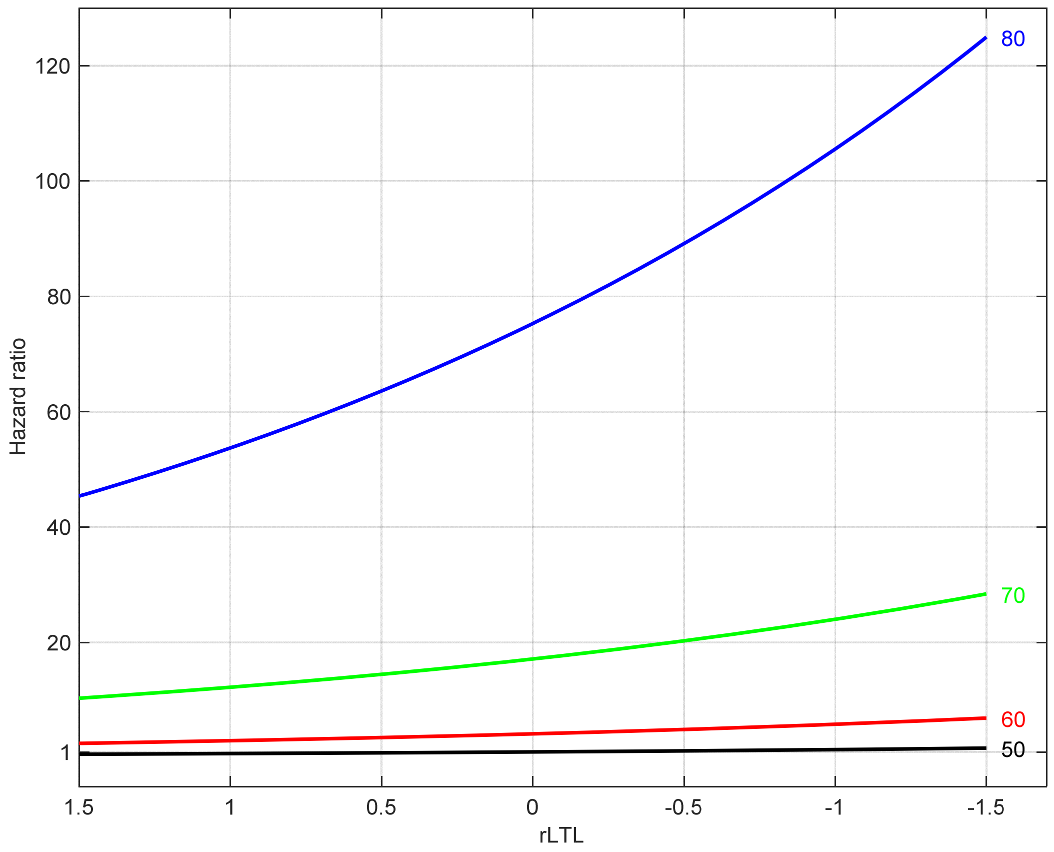

The figure is similar to eFigure 4 but it is for the model with stratified baseline hazards for age groups.

© 2020 Arbeev KG et al. *JAMA Network Open.*

eFigure 7. Hazard Ratios for Residual Leukocyte Telomere Length in Different Studies and Joint Analysis in the Model With Stratified Baseline Hazards

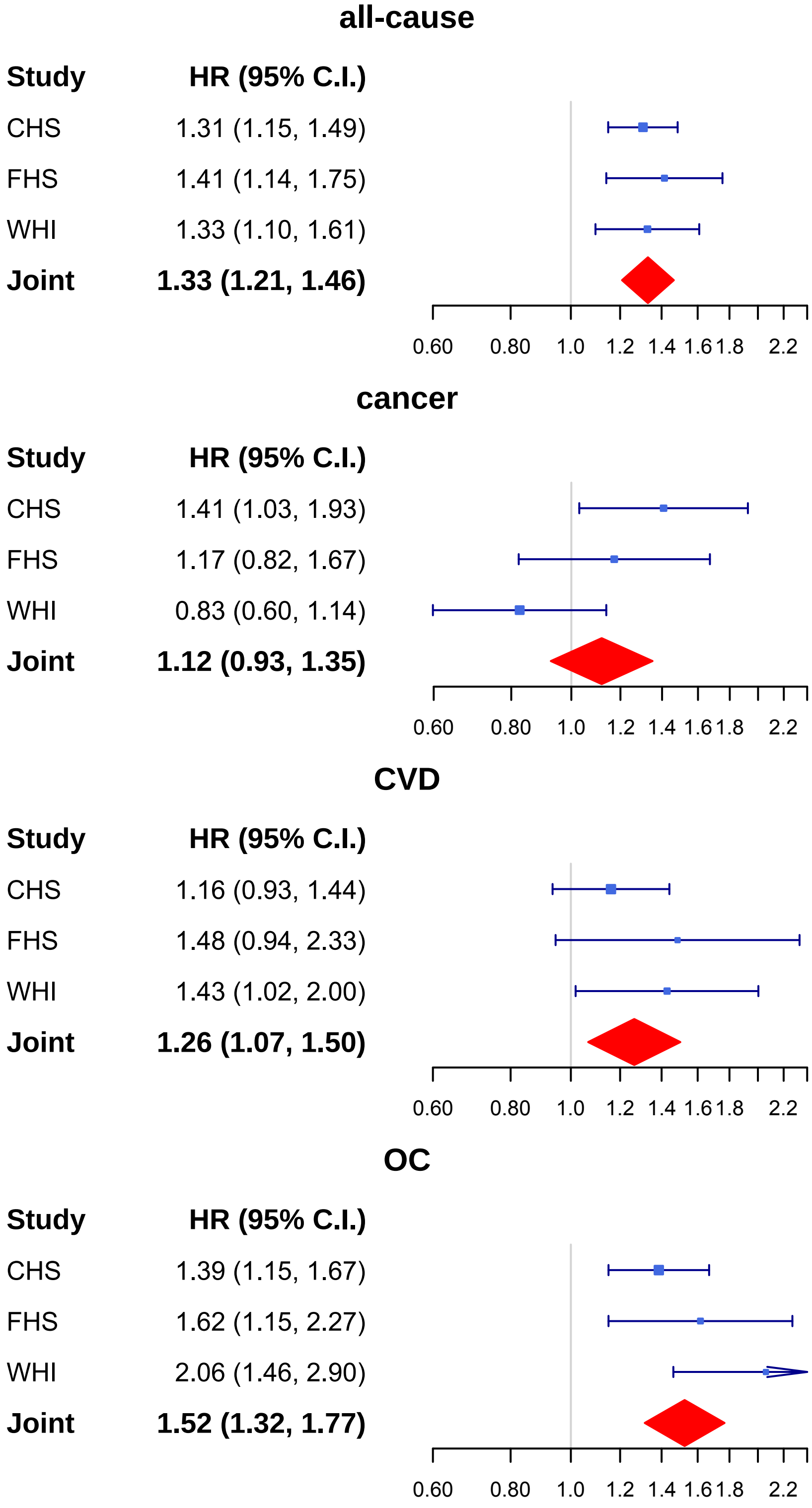

The figure is similar to Figure 4 in main text but it is for the model with stratified baseline hazards for age groups.

© 2020 Arbeev KG et al. *JAMA Network Open.*

eFigure 8. Hazard Ratios for Residual Leukocyte Telomere Length in Different Studies and Joint Analysis With Truncated Follow-up in the Model With Splines

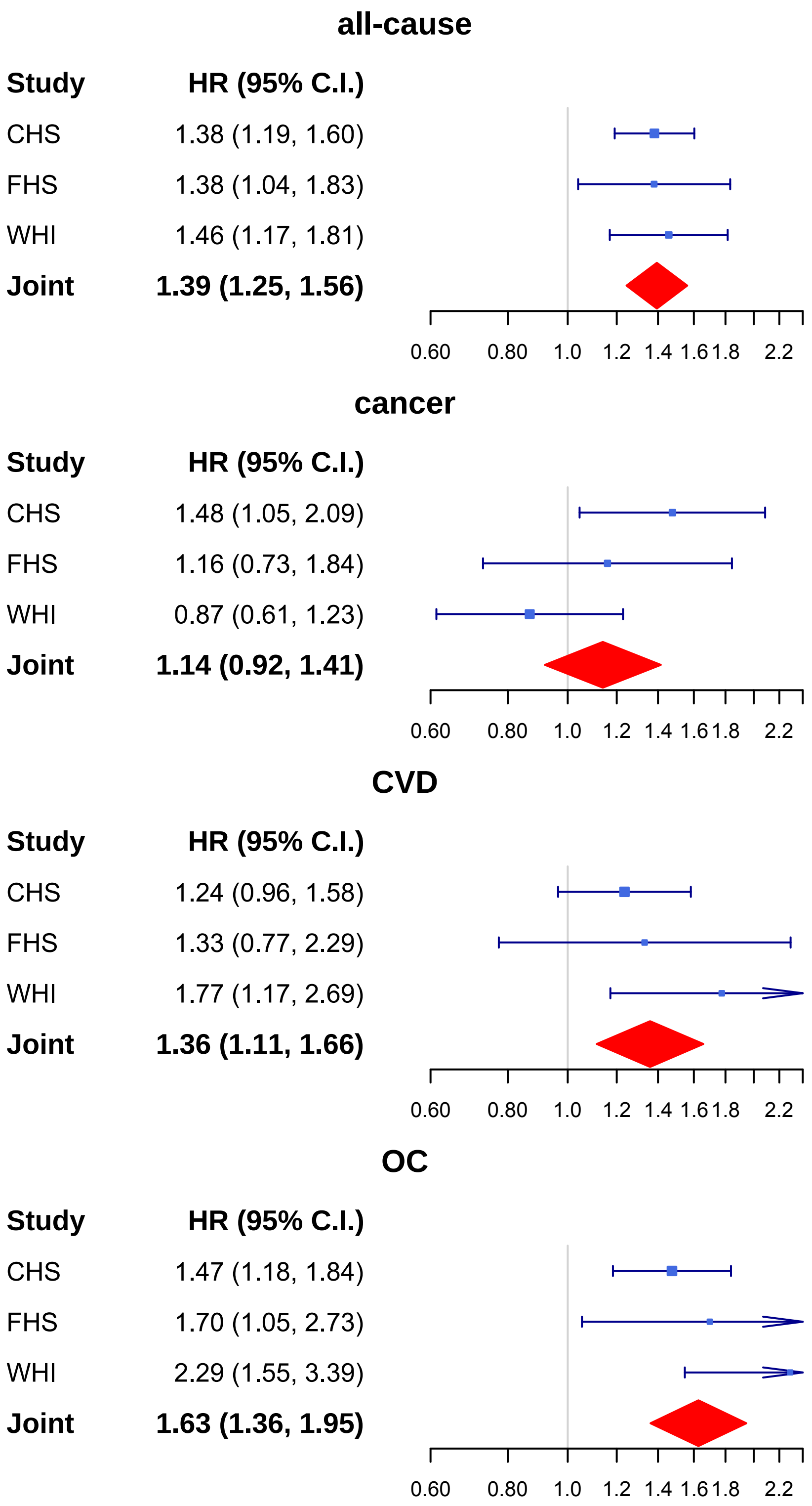

The figure is similar to Figure 4 in main text but it is for analyses with follow up truncated at 15 years in the model with splines for age.

© 2020 Arbeev KG et al. *JAMA Network Open.*

eFigure 9. Hazard Ratios for Residual Leukocyte Telomere Length in Different Studies and Joint Analysis With Truncated Follow-up in the Model With Stratified Baseline Hazards

all-cause

Study	HR (95% C.I.)
CHS	1.36 (1.18, 1.58)
FHS	1.36 (1.02, 1.80)
WHI	1.45 (1.17, 1.81)
**Joint**	**1.38 (1.23, 1.54)**

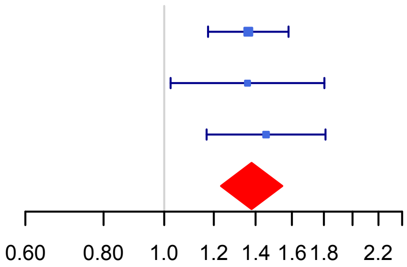

cancer

Study	HR (95% C.I.)
CHS	1.43 (1.01, 2.03)
FHS	1.14 (0.72, 1.81)
WHI	0.87 (0.61, 1.23)
**Joint**	**1.13 (0.91, 1.40)**

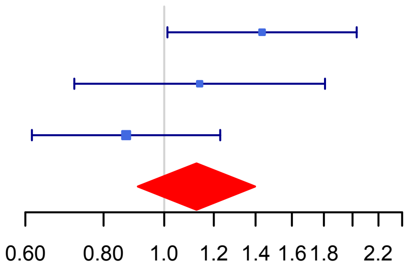

CVD

Study	HR (95% C.I.)
CHS	1.22 (0.95, 1.55)
FHS	1.31 (0.76, 2.26)
WHI	1.77 (1.18, 2.67)
**Joint**	**1.33 (1.09, 1.62)**

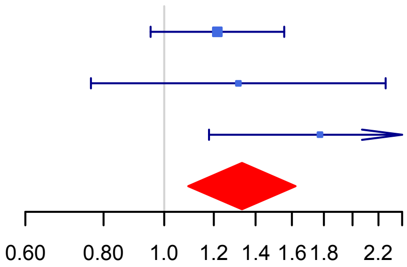

OC

Study	HR (95% C.I.)
CHS	1.46 (1.17, 1.82)
FHS	1.66 (1.03, 2.69)
WHI	2.28 (1.53, 3.38)
**Joint**	**1.62 (1.35, 1.93)**

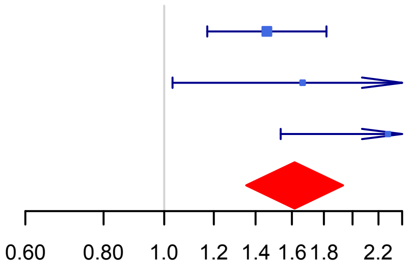

The figure is similar to eFigure 8 but it is for the model with stratified baseline hazards for age groups.

© 2020 Arbeev KG et al. *JAMA Network Open.*

eFigure 10. Density Plots of Leukocyte Telomere Length for Different Age Groups

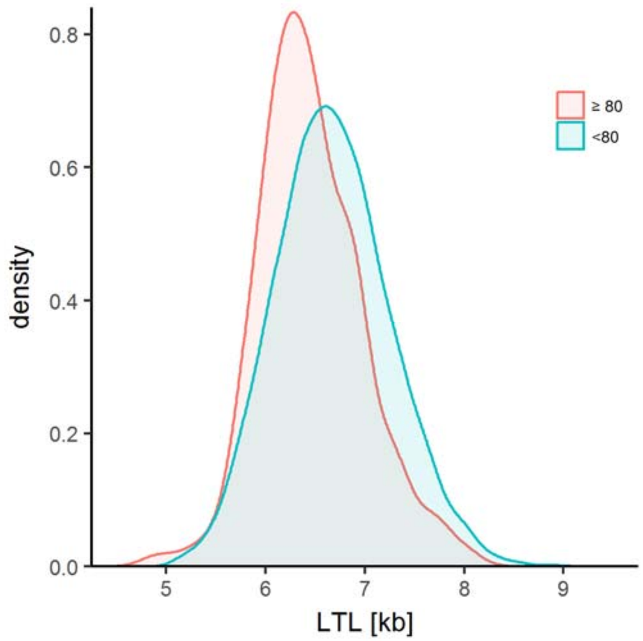

Smoothed histograms of LTL in respective age groups (age at blood draw ≥ 80 years and < 80 years) were generated by kernel density estimation. LTL is adjusted for sex and study.

© 2020 Arbeev KG et al. *JAMA Network Open.*
